# A NIR-responsive indocyanine green-genistein nanoformulation to control the polycomb epigenetic machinery for the efficient combinatorial photo/chemotherapy of glioblastoma†

**DOI:** 10.1039/c9na00212j

**Published:** 2019-04-16

**Authors:** Babita Kaundal, Anup K. Srivastava, Mohammed Nadim Sardoiwala, Surajit Karmakar, Subhasree Roy Choudhury

**Affiliations:** Institute of Nano Science and Technology, Habitat Centre Phase-10, Sector 64 Mohali Punjab India subhasreerc@inst.ac.in

## Abstract

Combinatorial photodynamics and chemotherapy have drawn enormous attention as therapeutic modalities *via* precise stimuli-responsive drug delivery for glioblastoma, which can overcome the limitations associated with conventional therapies. Herein, we have prepared an indocyanine green tagged, genistein encapsulated casein nanoformulation (ICG-Gen@CasNPs) that exhibits the near infra-red region responsive controlled release of genistein and enhanced cellular uptake in the human glioblastoma monolayer and a three-dimensional raft culture model *via* the enhanced retention effect. ICG-Gen@CasNPs, with the integrated photosensitizer indocyanine green within the nanoformulation, triggered oxidative stress, activating the apoptosis cascade, promoting cell cycle arrest and damaging the mitochondrial membrane potential, collectively directing glioblastoma cell death. The suppression of the polycomb group of proteins in the glioblastoma upon ICG-Gen@CasNPs/NIR exposure revealed the involvement of the epigenetic repression complex machinery in the regulation. Furthermore, ICG-Gen@CasNPs/PDT/PTT directed ubiquitination and proteasomal degradation of EZH2 and BMI1 indicates the implication of the polycomb in conferring glioblastoma survival. The increased activation of the apoptotic pathways and the generation of cellular reactive oxygen species upon inhibiting the expression of EZH2 and BMI1 strengthen our observations. It is worth noting that ICG-Gen@CasNPs robustly accumulated in the brain after crossing the blood–brain barrier, which represents the eminent biocompatibility and means that the system is devoid of any nonspecific toxicity *in vivo*. Moreover, a superior anti-tumor effect was demonstrated on a three-dimensional glioma spheroid model. Thus, this combinatorial chemo/photodynamic therapy revealed that ICG-Gen@CasNPs mediated epigenetic regulation, which is a crucial molecular mechanism of GBM suppression.

## Abstract 2

A novel ICG-Gen@CasNPs based chemo/photodynamic therapy showing proteasomal degradation of polycomb proteins and inducing oxidative stress mediated apoptosis in glioblastoma cells.

## Graphical abstract

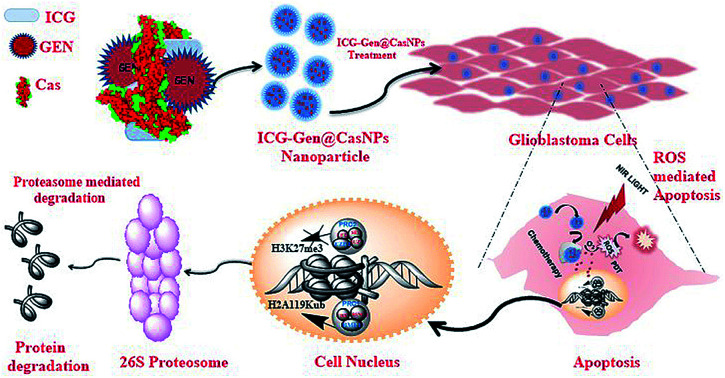

## 1. Introduction

Glioblastoma multiforme (GBM) is the most common primary malignant form of brain cancer and is aggressive, highly invasive and vascularized. Despite rigorous treatment modalities, the current prognosis and treatment methods are unfortunately very poor; with less than 5% of patients surviving five years after the initial diagnosis.^1,2^ The survival rates reported for conventional therapy following surgery, combined with radiation and chemotherapy, are dismal with a median reported survival of less than one year.^3,4^ Despite the plethora of research and technological advances that have been made in surgery and radio-chemotherapy, glioblastoma remains largely resistant to treatment. One of the major causes of extreme difficulty in the treatment of brain tumors is the presence of the blood–brain barrier (BBB), which hinders the availability of many cytotoxic drugs to the tumor.^5^ Presently, the alkylating agent temozolomide is being used clinically in chemotherapeutics against GBM.^6^ New approaches to provide an understanding of the molecular mechanism of glioblastomas and to design optimized therapies are thereby warranted.^7^ Genetics and epigenetic factors influencing GBM oncogenesis,^8^ with the over expression of transmembrane tyrosine kinase receptors, play a significant role in the progression of GBM and the activation of these receptors transduces signals which cause uncontrolled cell proliferation.^9^ The c-erbB gene encoding EGFR is amplified and overexpressed in up to 50% of malignant gliomas.^10^ The protein tyrosine kinase inhibitors reduce the uncontrolled growth of human glial cells by a mechanism that involves the inhibition of the epidermal growth factor receptor (EGF-R) associated tyrosine kinase.^11^

Genistein (5,7-dihydroxy-3-(4-hydroxyphenyl)-chrome-4-one) is a well-known inhibitor of the protein-tyrosine kinase (PTK), it attenuates cancer cell growth by inhibiting PTK-mediated signaling^10,12^ and is a poly-phenolic isoflavone, produced exclusively by the Leguminosae family.^13^ Genistein (Gen) is known for exhibiting a chemo-preventive effect^14^ in various cancers, it inhibits cellular oxidative stress, abrogates angiogenesis, induces apoptosis through upregulation of p21WAFI and Bax expression, downregulates MMP-9 and elicits TIMP-1 expression.^15–17^ Along with other pharmacological considerations, it suffers from a low aqueous solubility, low bioavailability and cleavage of the ring structure by the gut microflora.^18,19^ These limitations reduce the availability of Gen in the brain. Hence, increasing the passage of this drug through the BBB without toxic effects is a challenge for anti-glioblastoma therapy.^20^ Advances in nano-medicine have shown the potential of GBM therapy. Protein nanoformulation based drug delivery has wide applicability owing to the enhanced solubility, improved bioavailability, reduced toxicity and non-immunogenicity of nano-carriers.

Casein (Cas) is a milk protein and is recommended to be included in the daily diet, it is a renewable resource, is recognized as being safe and exerts characteristics such as non-antigenicity, biodegradability, an extraordinary binding capacity with various drugs, and is easy to scale up during manufacturing, which makes it a good carrier for conventional and novel drug delivery.^21^ Cas has advantages over other proteins as a matrix owing to its low cost, better amphiphilicity, good dispersibility, and because it undergoes rapid reconstitution in aqueous systems.^22^ Generally, a perfect drug nanocarrier system should have several characteristics including having a stable structure, high cellular uptake, high drug encapsulation efficiency, a desirable biodistribution, and reasonable pharmacokinetics, as well as exhibiting selective accumulation at the target site by the enhanced permeability and retention (EPR) effect.^23^ Recently, protein nanoparticles used as drug delivery vehicles and multimodal combinatorial approaches for photodynamic therapy (PDT) and photothermal therapy (PTT) have generated considerable interest.^23–26^ PDT is a well-established clinical treatment modality that involves light and chemical photosensitizers along with oxygen in the tissues for the successful treatment of various cancers and non-malignant diseases.^27^ An antitumor PDT using indocyanine green (ICG) has been reported previously.^28,29^ ICG is a hydrophilic tricarbocyanine near-infrared (NIR) emitting (650–850 nm) fluorophore that has been clinically approved by the FDA.^30^ ICG has a high tissue penetration and is used in diagnostic, NIR fluorescence image-guided oncologic surgery, fluorescence angiography, and for lymph node detection of various cancers.^31–33^ ICG produces singlet oxygen and heat upon NIR irradiation and is thus exploited as a therapeutic for PDT and PTT.^34^ However, it tends to disintegrate in aqueous solution, and this irreversible degradation can be accelerated by light irradiation and heat.^35^ Moreover, GBM heterogeneity resists single chemotherapeutics and combined multimodal therapies with multiple targets used in a synergistic way could emerge as a promising and effective therapeutic modality.^36^

Presently, the development of chemo/phototherapy remodeling epigenetic processes has gained significant interest. The administration of methyltransferase inhibitors in combination with PDT aid the effective release, enhancing antitumor immunity and potentiating the antitumor effects.^37^ The epigenetic regulator polycomb group (PcG) proteins play an important role in histone mediated epigenetic regulation which has been implicated in the malignant evolution of GBM.^38^ Polycomb genes (EZH2, RBBP7, SUZ12, and YY1) are specifically overexpressed in brain tumors.^39^ EZH2 is involved in some glioma cell processes; including proliferation, apoptosis, invasion, and angiogenesis. The histone methyltransferase enhancer of zeste homologue 2 (EZH2), is a methyltransferase component of the polycomb repressive complex 2 (PRC2), and deposits the histone mark H3K27me3, which promotes transcriptional repression and cell cycle regulation. During epigenetic gene silencing, PRC2 (EZH2) trimethylates the histone H3 at lysine 27 and recruits members of the polycomb repressive complex 1 (PRC1) which have E3 ligase activity for the monoubiquitination of histone H2A at lysine 119 which results in the transcriptional repression of the tumor suppressor genes. Therefore, multimodal targeting could trigger superior control over EZH2 and BMI-1 protein expression and emerge as a novel target for anti-GBM therapy.^40^

**Scheme 1:**
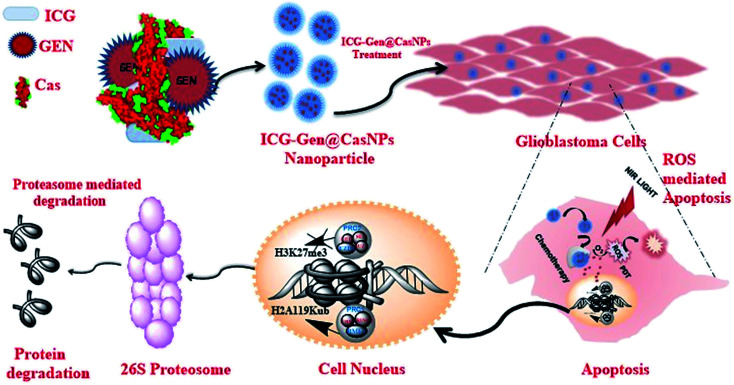
A schematic diagram representing ICG-Gen@CasNPs based chemo/photodynamic therapy directing the proteasomal degradation of the polycomb group of proteins, triggering oxidative stress to kill glioblastoma cancer cells.

Herein, we report (ICG-Gen@CasNPs) as a multimodal photodynamic and chemotherapeutic probe which has a superior anti-cancerous efficacy against glioblastomas. The developed nanoformulation consists of three main components, Cas nanoparticles as a nanocarrier prepared using the desolvation method, Gen as a multitargeted anti-cancerous drug and ICG as a NIR photosensitizer conjugated within. The nanoformulation exhibits acceptable morphological and structural features with robust photo-physical characteristics. The NIR responsive photosensitized triggered controlled drug release and a longer retention were observed inside the glioblastoma monolayer and the glioblastoma three-dimensional culture model. The NIR responsive glioblastoma cell death was positively correlated with apoptosis induction, and Sub-G1 cell cycle arrest and cellular reactive oxygen species (ROS) generation were implicated as the basis of the anti-cancerous effect. For the first time, the epigenetic regulation of the photo/chemotherapeutic potential of a nanoformulation was estimated using the reduced level of expression of PcG protein subunits. The nanoformulation induced proteolysis of EZH2 and BMI-1 resolved the molecular mechanism of the ubiquitination-mediated proteasomal degradation. Furthermore, the targeted depletion of BMI-1 and EZH2, which activated apoptosis and ROS generation confirmed PcG as a promising target in GBM for multimodal therapeutic approaches (Scheme 1). Finally, the ability of the ICG-Gen@CasNPs to cross the BBB was evident from the *in vitro* Transwell assay and the *in vivo* studies, and they robustly accumulated in the brains of mice. Furthermore, they induced negligible non-specific toxicity and a good biocompatibility. Therapeutic assessment using a three-dimensional glioma spheroid model revealed the therapeutic potential with strong regression. The results demonstrated a promising combinatorial anti-glioblastoma therapeutic method, clearly defining a novel epigenetic target for multimodal therapies.

## 2. Experimental section

### 2.1 Reagents

Casein protein was purchased from Sigma Aldrich (C3400). Acetone was purchased from Merck (1.00983.0511). MTT dye (TC191), glutaraldehyde 25% w/v solution (RM5927), propidium iodide (PI, TC252), streptomycin penicillin solution (A018), Dulbecco modified Eagle medium (DMEM, GIBCO, AL007S), fetal bovine serum (FBS, RM9955) and trypsin–EDTA were purchased from Himedia, as well as dialysis membrane 12 kD (D6191). The annexin V-PE/7-AAD kit (BD-Bioscience) and JC-1 cyanine dye (Abcam) were used for apoptosis and mitochondrial membrane potential analysis *via* fluorescence-activated cell sorting (FACS) respectively. DAPI (Himedia) was used for nuclear staining and 2′7′-dichlorofluorescein diacetate (Himedia) was used as a fluorescent dye for ROS detection respectively.

### 2.2 Synthesis of placebo casein nanoparticles

Casein nanoparticles (CasNPs) were prepared using the desolvation method by adding acetone dropwise under a constant pH of 8.2 and stirring at 1800 rpm at room temperature (25 °C). The prepared CasNPs were further cross-linked using 8% glutaraldehyde solution in water at 1.175 μl mg^−1^ of protein, and the solution was kept on a rotary shaker overnight. The acetone was evaporated using rotary evaporation, leaving the nanoparticles suspended in the water phase, this was centrifuged three times at 30 000 × *g* for 30 min for complete removal of the unbound glutaraldehyde. Furthermore, after the purification process, the CasNPs were freeze-dried in a lyophilizer. The optimization of the best reaction conditions was carried out using Box–Behnken design (BBD) on Design Expert software (Stat-Ease Inc. Minneapolis, USA). A total of 17 experimental trials were recorded using the design (Table S1†) after consideration of the Cas concentration, the rate of magnetic stirring and the percentage of solvent (acetone) as independent variables in three levels; low (−1), medium (0) and high (+1). The particle size (nm) and polydispersity index (PDI) of the CasNPs were selected. The quadratic second-order model-based fitting with ANOVA provided the coefficient of correlation (*r*_2_), which was further analyzed using the numerical desirability function and graphical optimization methods.^41^

### 2.3 Genistein encapsulation and ICG tagging

Genistein was dissolved in a minimal amount of acetone and added dropwise to the 0.5% Cas solution at a mass ratio of 1 : 6 respectively. The reaction was stirred for 2 h at room temperature and then acetone was added dropwise as described in the synthesis of the placebo CasNPs. After washing three times and purification, the ICG-Gen@CasNPs were freeze-dried and lyophilized. ICG was dissolved in water at a mass ratio of 1 : 30 and added to the prepared nanoparticles and left overnight for tagging.

### 2.4 Drug loading and drug encapsulation efficiency

The amount of Gen in the supernatant obtained after the drug loading studies was determined using a HPLC system (Waters) equipped with a UV detector with an injection volume of 20 μl using a C18 column (water spherisorb, 250 × 4.6 mm, 5 μm). The mobile phase was composed of methanol/distilled water (95/5%) at a flow rate of 1 ml min^−1^ and a detection wavelength of 261 nm. The retention time was 2.87 ± 0.38 min. Calibration curves for Gen (correlation coefficient was 0.992) at concentrations varying from 0.2 to 0.6 mg ml^−1^ were used for the analysis. A drug loading efficiency of 10% with a drug entrapment efficiency of 98.95% were calculated using the formulas given below:
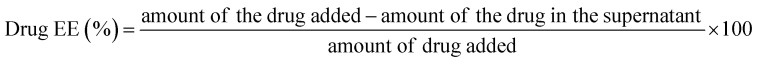

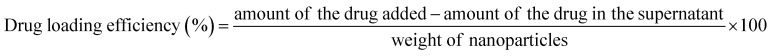


### 2.5 Characterization of casein nanoparticles and drug loaded casein nanoparticles

The hydrodynamic diameter, particle size distribution, and zeta potential measurements were performed using a Malvern particle size analyzer based on a backscattering angle of 173°. Transmission electron microscopy (TEM) images were obtained on a JSM 2100 operated at 120 kV. Circular dichroism (CD) spectra were recorded using a JASCO J-1500 circular dichroism spectrophotometer, Easton, MD, USA, using demountable cells (0.1 mm path length, Hellma). A high performance liquid chromatography (HPLC) Waters system was used to make a standard for the drug and calculate the drug loading efficiency using Breeze 2.0 analysis software. The interaction of Gen with Cas was studied using a UV-vis spectrophotometer. Fourier transforms infrared (FTIR) spectra were recorded on a Cary Agilent 660 IR spectrophotometer. For each spectrum, 256 scans and a 4 cm^−1^ resolution were applied over the range of 400–4000 cm^−1^. X-ray diffractometry (XRD) was recorded on a Bruker powder XRD D8 X-ray diffractometer. For the powder XRD analysis, the samples were first lyophilized prior to analysis. XRD analysis was performed from the 2θ values of 2–80° with a scan rate of 0.2 scans per minute using powder XRD. A microtiter plate reader BioTek Synergy H1, Finland, was used for the measurement of the absorbance and fluorescence emission spectrum, revealing the physical interaction between ICG/Gen and the CasNPs. Cas (1 mM) was prepared in Milli-Q water, Gen stock was prepared in methanol diluted in Milli-Q water for the interaction studies and a confocal laser scanning microscope (Leica Microsystems) was used for the fluorescence imaging.

### 2.6 *In vitro* drug release studies

Drug release studies were performed in a 20% ethanolic solution of phosphate buffer saline (PBS) at pH 7.4 using a 12 kD cutoff dialysis membrane. 1 mg of Gen was weighed and suspended in 1 ml of the 20% methanol solution of PBS at pH 7.4, the respective amount of drug loaded CasNPs were dissolved in the same solvent. The Gen release was analyzed in 9 ml of 20% ethanolic solution of PBS pH 7.4 as a releasing media at 37 °C with stirring at 150 rpm in the presence and absence of light. A 1 ml aliquot was taken at different time intervals and the sink was replaced with the same amount of solvent, the aliquot was analyzed using a HPLC Waters system. The drug solubility study was performed in 1× PBS pH 7.4 with 0.5 mg ml^−1^ of Gen, and an equal ratio of ICG-Gen@CasNPs respectively. The absorbance of the drug was measured every 24 h up to 72 h using a UV-vis spectrophotometer. A stability study was performed mimicking different physiological conditions of DMEM and DMEM + 10% FBS, water, PBS for ICG-Gen@CasNPs and placebo CasNPs; the variations in the particle diameter and zeta potential were analyzed using a Malvern particle size analyzer.

### 2.7 Cell culture

Human glioblastoma cell lines LN18, C6 and HEK293T were purchased from the National Cell Repository NCCS Pune, India and maintained in cell culture medium DMEM supplemented with 10% FBS and 1% antibiotic solution at 37 °C under 5% CO_2_.

### 2.8 Cellular uptake studies using confocal laser scanning microscopy

The cells were seeded in a 6-well plate on coverslips with a density of 5 × 10^4^ cells per well in 2 ml of culture medium and cells were seeded at 2 × 10^5^ in a cell hanging membrane for raft preparation. The LN18 cells were seeded at a density of 3 × 10^5^ in 1% agar-coated 12-well plates containing DMEM supplemented with FBS (10%) and incubated at 37 °C (95% humidity, 95% air, and 5% CO_2_) and cultured for 3–5 days, until spheroids were formed. Half of the culture medium was replaced with fresh medium twice a week. After round spheroids had formed those with a 200 μm diameter were collected, transferred and cultured in agarose-coated (0.1%) chamber slides with the same culture medium. The cells, rafts, and spheroids were incubated with rhodamine B tagged nanoparticles at a concentration of 25 μg ml^−1^ for 24 h. The cellular uptake and distribution of the CasNPs were investigated using a confocal laser scanning microscope (LSM880 Zeiss System).

### 2.9 Optimization of NIR light and photothermal experiments in solutions

Cells were seeded in 24 well plates at a density of 5 × 10^4^ on the day prior to the experiment. Once cells formed a monolayer they were exposed to NIR light at different time intervals at a fixed distance of 25 mm and a generating power of 0.6 W cm^−2^ for variable time periods starting from 2–3 min with 2 s interval time of exposure. Morphological characterization was performed after 2 and 24 h of light exposure using an optical microscope and the IC_50_ value was determined using the MTT assay. The aqueous solutions of PBS, placebo CasNPs, ICG, GEN ICG-Gen@CasNPs (1 mg ml^−1^) were placed in a quartz cuvette and irradiated using an 808 nm laser at a power density of 0.6 W cm^−2^ for 15 min to determine whether 200 μg ml^−1^ was sufficient to increase the temperature. The laser power density was measured using a laser power meter.

### 2.10 Cytotoxicity assay and photodynamic therapy

The proliferation of LN18, C6 and HEK293T cells under various treatment conditions were evaluated by using the colorimetric MTT assay at 570 nm using a microplate reader. Photodynamic therapy was performed using NIR light. Cells were seeded in 24 well plates at a density of 1.5 × 10^4^ on the day prior to the experiment. Once the cells formed a monolayer, they were treated with different concentrations of ICG-Gen@CasNPs, placebo CasNPs, and the drugs Gen and ICG for 4 h in a 2% FBS in DMEM media. After this NIR light (0.6 W cm^2^) was exposed at different time intervals at a fixed distance of 25 mm for variable time periods starting from 1–3 min with a 2 s interval time of exposure on a NIR light treatment plate and then incubated overnight. The MTT assay was performed at 48 h with the control which was untreated and with NIR light treatment. The results were presented as a percentage compared to the control using at least three independent experiments in a triplicate format for each treatment.

### 2.11 Measurement of ROS production

Changes in the ROS level of the ICG-Gen@CasNPs treated glioblastoma cells were determined using staining with the fluorescent marker 2′,7′-dichlorodihydrofluorescein diacetate (DCFH-DA). Briefly, the LN18 cells were pretreated with 10 mM DCFH-DA for 1 h prior to treatment with ICG-Gen@CasNPs, placebo CasNPs, and the drugs Gen and ICG with and without light (48 h). Then, the intensity the of fluorescence was measured using a Microtiter plate reader Biotech Synergy H1, Finland.

### 2.12 Cell cycle analysis

Propidium iodide staining was performed to determine the distribution of cells in different phases of the cell cycle using flow cytometry analysis. Briefly, LN18 cells were seeded into 6-well plates at a density of 1 × 10^5^ cells/well. The cells were treated with ICG-Gen@CasNPs, placebo CasNPs, and the drugs Gen and ICG with and without NIR light treatment. The cells were collected by centrifugation, and then fixed and stained with a PI solution at 4 °C for 30 min in the dark to analyze the cell cycle distribution based on the DNA content of cells using a flow cytometer. Data analysis was performed using the FlowJoV10 software.

### 2.13 Annexin V apoptosis assay

Apoptosis detection was performed using the FITC annexin V apoptosis detection kit (BD Biosciences, USA). LN18 cells at a concentration of 8 × 10^4^ cells/ml were seeded and treated with the placebo CasNPs, ICG-Gen@CasNPs, ICG and Gen for 48 h with and without NIR light exposure. The cells were harvested, washed with PBS, resuspended in 1× annexin V binding buffer, and stained with annexin V and PI for 15 min at room temperature in the dark. Apoptosis was detected using a flow cytometer. Distribution of the cell population in different quadrants was analyzed with quadrant statistics. The lower left quadrants consist of viable cells, the lower right quadrants early apoptotic, and the upper right quadrants late-apoptotic or necrotic cells.

### 2.14 Analysis of mitochondrial transmembrane potential (Δ*Ψ*_m_)

A stock solution of JC-1 (1 mg ml^−1^) was prepared in DMSO and freshly diluted with the assay buffer. Briefly, LN18 cells (5 × 10^4^) were seeded in six-well plates and allowed to attach overnight. The cells were treated with CasNPs, ICG-Gen@CasNPs, ICG and Gen, with and without light as described above. The cells were collected by trypsinization and washed with PBS and incubated for 15 min at 37 °C in a medium containing 10 μg ml^−1^ JC1. The cells were washed with PBS, resuspended in 0.5 ml PBS, and analyzed using a flow cytometer.

### 2.15 Real-time quantitative PCR analyses

Real-time quantitative PCR (qPCR) was used to validate the gene expression in each cell model before and after treatment with the placebo CasNPs, drug loaded CasNPs, and the drugs ICG and Gen for 24 h. The cells were also treated with the EZH2 inhibitor (EPZ011989) and BMI-1 inhibitor (PRT4165). Briefly, the total RNA was extracted from the treated cells using a RNeasy extraction kit (Applied Bioscience, Thermo Fischer) and the RNA concentrations were determined using a BioSpec-nano UV-vis spectrophotometer (Shimadzu, Columbia, MD). Reverse transcription was carried out using a high-capacity cDNA reverse transcription kit (Thermo Scientific) according to the manufacturer's protocol. Following cDNA preparation, 100 ng of each sample was loaded into the 96 well qPCR plate at a final volume of 20 μL, using GAPDH as the endogenous control. Taqman primers for all gene targets were used for cDNA template amplification. The qPCR plate was read using an Applied Biosystems Quantstudio 3 qPCR system (Thermo Scientific) with an initial cycle step of 50 °C for 2 min followed by 40 cycles of 95 °C for 15 s then 60 °C for 1 min. The cycle threshold (*C*_t_) values were automatically determined by the software, and all samples were performed in triplicate for statistical analysis.

### 2.16 Western blot analysis

The glioblastoma cells were treated for 48 h with different concentrations of placebo CasNPs, ICG-Gen@CasNPs, the drugs Gen and ICG, and the EZH2 inhibitor. The BMI-1 inhibitor and the whole-cell protein lysates were prepared using RIPA buffer followed by quantitation of protein using the Bradford reagent (Himedia). Cell lysates with 25 μg of protein were separated using sodium dodecyl sulfate-polyacrylamide gel electrophoresis (SDS-PAGE), transferred to polyvinylidene fluoride (PVDF) membranes, and blocked with 5% BSA probed with the respective primary and secondary antibodies. The binding of the antibody was visualized using an ECL substrate (BIO-Rad) and this was followed by detection using a ChemiDoc XRS+ imaging system with image lab software (Bio-rad). Anti-mouse and anti-rabbit antibodies were purchased from either Cell Signalling Technology (Danvers, MA, USA) or Santa Cruz Biotechnology (Santa Cruz, CA, USA). To detect the association of the proteasome pathway, glioblastoma cells were treated with MG132 for 4 h followed by treatment of ICG-Gen@CasNPs for 48 h along with the MG132 untreated group and were processed *via* the protocols mentioned above.

### 2.17 Detection of ubiquitinated PcG proteins

A/G beads were used for the ubiquitination assays, cell lysates of protein were immunoprecipitated with anti-IgG, anti-Bmi-1, and anti-EZH2 with A/G beads SCBT (sc-2003) and shaken overnight at 4 °C. The precipitated A/G beads were washed twice with PBS and electrophoresed using SDS-PAGE before being transferred to PVDF membranes, blocked with 5% BSA, and probed with the respective primary ubiquitin antibody and secondary anti-rabbit antibodies. The binding of the antibody was visualized using ECL substrate (BIO-RAD) and this was followed by detection through a ChemiDoc XRS+ imaging system with image lab software (Bio-rad).

### 2.18 Trans-well model and permeability assay

The mouse primary endothelial cells were grown on type I collagen (BD Biosciences)-coated 6.5 mm Transwell culture inserts with a pore size of 0.45 lm (Corning Life Sciences) for 78 h until a confluent multilayer was established. The permeability test was performed by adding a particular concentration of rhodamine tagged nanoparticles to the upper chamber (luminal) and aliquots were collected from the lower chamber (abluminal) and measured and quantitated by measuring the fluorescence intensity on a Tecan MPlex 200 pro microplate reader (excitation at 532 nm and emission at 620 nm).

### 2.19 *In vivo* and *ex vivo* bio-distribution of nanoparticles

The animal experiments were performed as per the requirements and guidelines of the Committee for the Purpose of Control and Supervision of Experiments on Animals (CPCSEA), Government of India, and were approved by the Institutional Animal Ethical Committee. In order to assess the tissue distribution in a pre-clinical model, the BALB/c mice were injected *via* the tail vein with 200 μl of 5 mg ml^−1^ ICG-Gen@CasNPs and the photoluminescence (IVIS spectrum) of the ICG was observed for 4 h at a time interval of 30 min. The animals were euthanized as per ethical guidance after 4 h and dissected, the kidney, spleen, liver, brain and heart were collected. For a clearer distribution of the ICG-Gen@CasNPs, the isolated organs were imaged *ex vivo*. Tissues were then embedded at the optimal cutting temperature (OCT) and kept frozen at −80 °C until needed. The frozen tissues were sliced into 6 μm thick sections and mounted onto glass slides. The tissue sections were thawed at room temperature for 30 min and the fluorescence intensity was measured using a C600 imager (Azure System). The images were analyzed using the public-domain Image J software (US National Institutes of Health, Bethesda, MD). The obtained fluorescence intensity was normalized for each tissue type using the same threshold settings. Data are reported as the average channel fluorescence of the tissue, given as relative units after background subtraction with the untreated control. For visual illustrations of the fluorescence signals, a histogram was drawn representing the quantitative results.

### 2.20 Therapeutic assessment on glioma 3D spheroids

The monolayer LN18 spheroids grown on agarose chamber slides were treated with rhodamine tagged CasNPs. After 24 h the plates were washed with PBS twice and the spheroids were fixed with 4% PFA and washed with PBS. Nuclei were stained with DAPI, mounted on glass slides and imaged using confocal microscopy (LSM Zeiss 880). The software was used to obtain confocal z-stacks for the spheroids. Images were taken every 20 μm down through the multilayer, visualizing tumor cells in the individual layers.

### 2.21 Statistical analysis

All data were analyzed using the statistical package origin 8.5 version of the software. Two-way ANOVA was performed to analyze the data from different groups, and Tukey's test was performed for further comparison of the groups, a *p*-value of less than 0.05 was considered to be statistically significant.

## 3. Results and discussion

### 3.1 Synthesis and characterization of placebo CasNPs and ICG-Gen@CasNPs

**Fig. 1:**
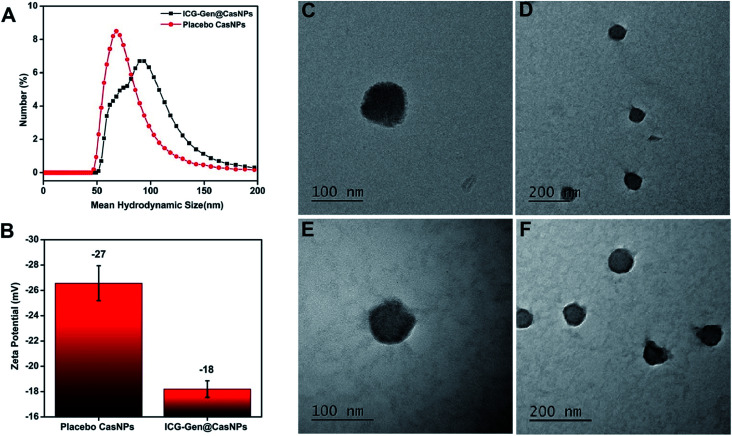
The mean hydrodynamic diameter distribution of the placebo CasNPs and ICG-Gen@CasNPs are 68 ± 10 nm and 93 ± 7 nm respectively (A); the negative zeta potential of placebo CasNPs and ICG-Gen@CasNPs are represented in the histogram (B); morphological characterization and TEM micrographs of the placebo CasNPs (C, D) and the ICG-Gen@CasNPs (E, F) are shown.

ICG-Gen@CasNPs were constructed using the desolvation method by following the standard protocols.^42^ The effect of the Cas concentration, magnetic stirring, and acetone concentration were monitored and controlled to obtain a narrower size distribution for the placebo CasNPs, and ICG-Gen@CasNPs. The variation among the particles size and PDI as a function of the stirring rate, acetone concentration and Cas concentration are represented as a 2D contour plot and 3D response surface plots (Fig. S1†). The 2D contour plots suggested that the magnetic stirring rate and protein concentration are the major variables inducing the variation in the particle size and the PDI significantly. Whereas the complex interaction of the variables in the 3D response plots confirm that the effect of the protein concentration and the stirring rate are crucial determinants for the desirable particle diameter and PDI. The numerical optimization and desirability function provides the best-optimized amount of the variables. A higher concentration of protein (1), the maximum stirring rate (1800 rpm) and acetone (100%) provide the CasNPs with an average diameter of 127.1 nm with a PDI of 0.35 (Table S1†). The observed value for the prepared CasNPs was somewhat closer to the predicted response (with the error ± 10%). The loading efficiency of the ICG-Gen@CasNPs was 10.2% and the mean hydrodynamic diameter were found to be around 68 ± 10 nm for the placebo CasNPs, and 93 ± 7 nm for the ICG-Gen@CasNPs respectively, obtained *via* dynamic light scattering (DLS) with a monodispersed solution phase indicated by the PDI of 0.129 ± 0.10 nm and 0.182 ± 0.15 nm for the placebo CasNPs and ICG-Gen@CasNPs, respectively (Fig. 1A). In addition to the diameter, the surface zeta potential of the placebo CasNPs and ICG-Gen@CasNPs were −26.56 ± 8 mV and −18.2 ± 9.5 mV, respectively (Fig. 1B), this ensures the better dispersibility and stability of the nanoparticles in an aqueous environment. The morphological features of the placebo CasNPs Fig. 1C and D and the ICG-Gen@CasNPs Fig. 1E and F were obtained using TEM and revealed a uniform spherical morphology with a mean observed diameter of 100 nm. The slight variation in the diameter observed using DLS and TEM could be due to the principally different observation and sample preparation steps. Nanoparticle size is an important parameter for cellular uptake, targeted delivery and the anticancer efficacy of nanomedicines in the treatment of glioblastoma.^43^ Therefore, our results demonstrated that the prepared ICG-Gen@CasNPs nanoparticles are suitable for the treatment of GBM as they have a size that is less than 100 nm, which is needed for anti-glioma activity and permeability to cross the BBB.

### 3.2 Drug and nano-carrier interactions and compatibility

**Fig. 2:**
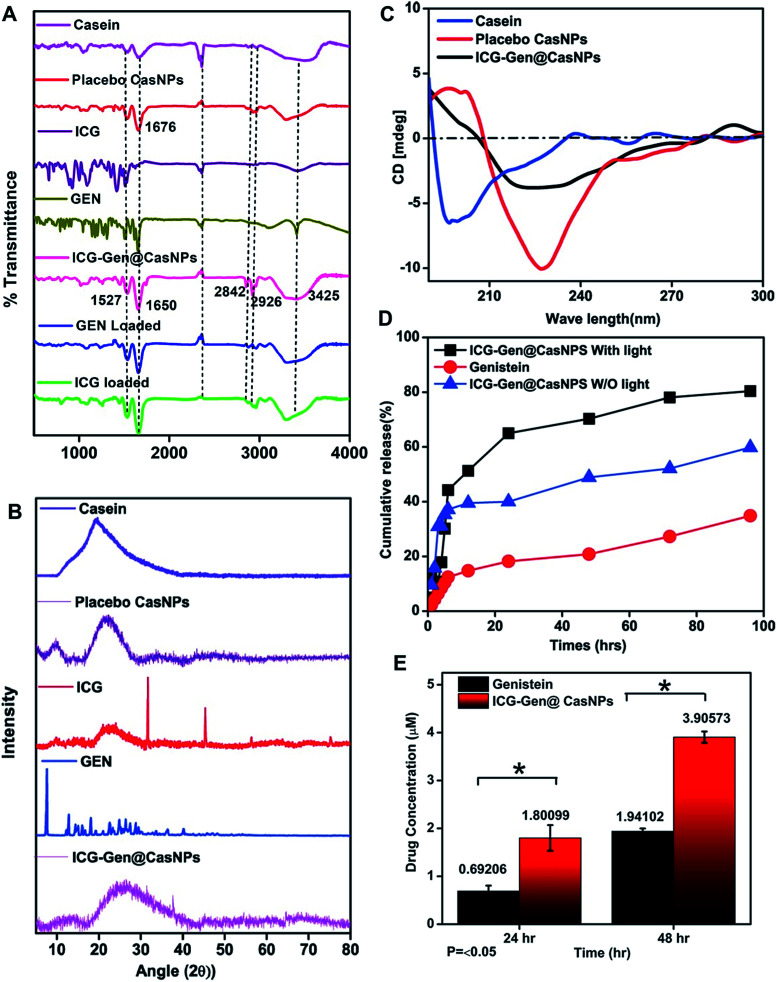
The FTIR spectra of the placebo CasNPs (red), ICG loaded (green), Gen loaded (blue), Cas (magenta), ICG-Gen@CasNPs (dark pink), free Gen (green), and free ICG drug (purple) from 500 to 4000 cm^−1^ (A); XRD patterns for ICG, Gen, ICG-Gen@CasNPs, placebo CasNPs, and Cas (B); CD spectroscopy showing the secondary structure of Cas, placebo CasNPs and ICG-Gen@CasNPs (C); *in vitro* percentage cumulative drug release profiles for Gen and ICG-Gen@CasNPs with NIR light and without NIR light (D); and solubility profiles for Gen and ICG-Gen@CasNPs (E).

To determine the interactions and chemical modifications occurring during the synthesis process FTIR analysis was conducted for Cas, the placebo CasNPs, ICG@CasNPs, Gen@CasNPs, ICG-Gen@CasNPs, ICG and Gen. The amide II (N–H bending vibration and C–N stretching vibration) and amide I peak (C=O stretching vibration of the amide group coupled to the in-phase bending of the N–H bond and the stretching of the C–N bond) were considered to study the protein secondary structure and an altered transmittance and a shift of 1673 cm^−1^ and 1525 cm^−1^ were observed among the Cas, CasNPs, ICG@CasNPs, Gen@CasNPs and ICG-Gen@CasNPs, indicating a crucial interaction at the respective functional group. The shifting of the amide I peak towards a lower wavenumber observed at 1661 cm^−1^ in the ICG-Gen@CasNPs revealed the interaction of ICG/Gen with Cas during encapsulation. Other important peaks at 2842 cm^−1^ (CH_2_ symmetric stretch), 2936 cm^−1^ and 3425 cm^−1^ (–OH stretching) (Fig. 2A) were consistent in the ICG-Gen@CasNPs and their absence in others clearly demonstrated the interaction of ICG/Gen with the CasNPs. The functional group analysis revealed a non-covalent interaction among the three-component system which critically determines the complete bioavailability at the target site. The crystallinity and phase of Gen and ICG in the powdered state displayed crystalline characteristic diffraction peaks at 7.4, 12.9, 18.15, 25.2, 36.1, and at 31.5, 45.4, 5.6 for the 2θ value using XRD respectively. Although Cas, the placebo CasNPs, and ICG-Gen@CasNPs showed almost amorphous characteristics, supporting the complete entrapment of Gen and ICG in the CasNPs (Fig. 2B). The CD spectroscopy further revealed the conformational transition of the protein secondary structure of Cas originating from the alpha-helix turns to a beta sheet and the random coils in the CasNPs, indicating the degree of protein aggregation and denaturation constituted by the CasNPs morphology, stability, and functionality (Fig. 2C). The CD data gave information about the mechanism of nanoparticle synthesis *via* the desolvation method and the alpha helix was converted to the beta sheet and random coil structure showing the formation of the nanoformulation. Interestingly, confirmation of the Gen/ICG and Cas interaction was observed using UV spectral absorbance and steady-state tryptophan fluorescence emission intensity measurements. Cas (1 mM) with varying concentrations of 10, 20, 30, 40, and 50 μM Gen and ICG showed an increase in the absorbance (*λ*_max_) between 230–300 nm, primarily indicating it was devoid of any physical covalent interactions between the Cas, Gen and ICG (Fig. S2a and c†). Although, the quenching of the steady-state tryptophan fluorescence maxima of 1 mM of Cas positively correlated with the increasing concentration of Gen, ICG demonstrated noncovalent interactions with Cas in the aqueous phase (Fig. S2b and d†). Moreover, the UV-vis absorbance of the ICG-Gen@CasNPs showed the featured absorbance peak for Gen and ICG, confirming the presence and crucial interaction in the formation of the nanoformulation (Fig. S3†). These results indicated the crucial non-covalent interactions among the nanoformulation components which endow the nanoformulation with a superior photo/aqueous stability in intravenous applications.

### 3.3 *In vitro* release and the solubility and stability profile of ICG/Gen

The *in vitro* drug release of the ICG-Gen@CasNPs was evaluated at pH 7.4 with and without NIR light exposure. Remarkably, both the ICG-Gen@CasNPs with and without NIR light exposure released the drug more strongly at pH 7.4 (Fig. 2D) as compared to the drug Gen. The cumulative release (%) of the ICG-Gen@CasNPs without NIR light was approximately 60% and with NIR light was approximately 80% for up to 96 h respectively. ICG, as a photosensitizer, triggers a superior drug (Gen) release profile under stimulation with NIR light. The solubility profile of the Gen and ICG-Gen@CasNPs in PBS indicated a significant increase in the drug solubility up to 48 h, indicating that the ICG-Gen@CasNPs nanoformulation overcomes the aqueous solubility problem of Gen (Fig. 2E). The bio-stability study shows the compatibility of the ICG-Gen@CasNPs in various physiological fluid conditions which were very promising for further biological applications. The zeta potential measurement showed that they were devoid of any significant changes up to a duration of 48 h in DMEM and DMEM + 10% FBS, water and PBS (Fig. S4†). The data collected on the characteristics confirm the suitability of the prepared nanoformulation for future therapeutic assessment.

### 3.4 NIR induced *in vitro* combination chemo/photo-dynamic therapy

**Fig. 3:**
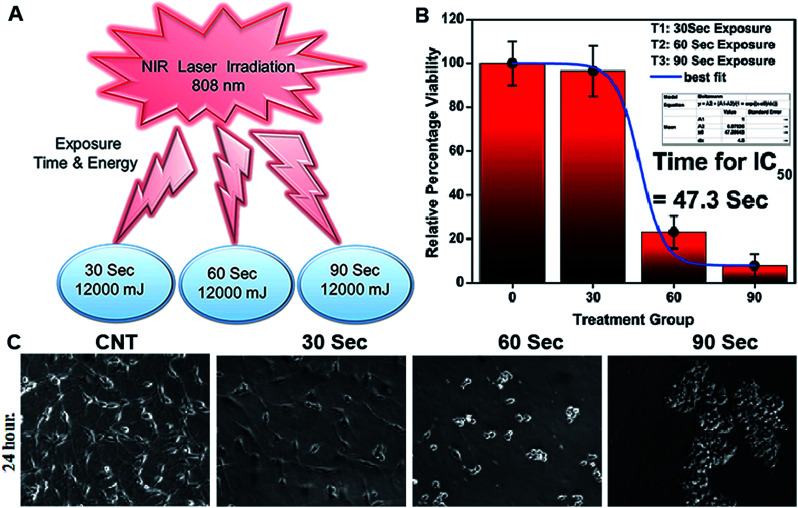
Exposure to NIR light at different time intervals and different power exposures was undertaken (A); IC_50_ of light exposure (B); and morphology characterization after 24 h of light exposure using an optical microscope (C).

**Fig. 4:**
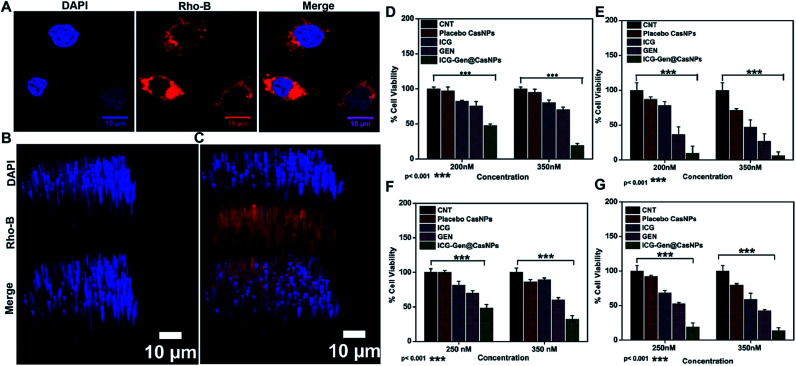
Cellular uptake of rhodamine B tagged placebo CasNPs in the LN18 cell line after 4 h (A) using confocal light scanning microscopy; uptake study showing the 3D raft of LN18 using confocal light scanning microscopy for the control (B) and placebo CasNPs (C); cellular viability assay shown in LN18 cells with different doses for the control, placebo CasNPs, Gen, ICG, ICG-Gen@CasNPs without NIR light (D) and with NIR light (E); and in C6 without NIR light (F) and with NIR light exposure (G).

NIR light exposure optimization was performed for different time intervals of 1–3 min at a fixed distance of 25 mm and an optimized power of 0.6 W cm^−2^ (Fig. 3A and B). The results of the pulse exposure revealed that 1 min light exposure did not result in the death of any cells or a change in the morphology of the growing untreated cells, and these parameters were used for further experiments. Utilizing an MTT [3-(4,5-dimethylthiazol-2-yl)-2,5-diphenyltetrazolium bromide] based cellular viability assay, the NIR light exposure time duration of 47.3 s was calculated as the IC_50_ for the LN18 glioma cell line (Fig. 3C). To assess the therapeutic efficacy of the nanoformulation, the cytotoxic activity of the ICG-Gen@CasNPs, placebo CasNPs, Gen and ICG suspensions were evaluated using a MTT assay against the LN18 and C6 GBM stem cell lines under NIR exposure and without NIR light. The calculated and reported IC_50_ value for Gen was found to be 40 μM in the LN18 cell line up to 48 h. The 250 nM ml^−1^ and 350 nM ml^−1^ dose of Gen in the ICG-Gen@CasNPs showed an enhanced cytotoxicity with a NIR exposure of 30 s, compared with comparable doses of Gen, ICG, and placebo CasNPs in LN18 cells at approximately 10% (Fig. 4D and E) and in C6 cells at approximately 20% (Fig. 4F and G) cell viability. The results show that our nanoformulation ICG-Gen@CasNPs at a 350 nM dose are more potent compared to the reported value for 40 μM IC_50_ of Gen^44^ and demonstrate increased death of the GBM cells with NIR light exposure *via* the generation of ROS as reported in previous reports.^45^ These results demonstrated that the smaller size of the ICG-Gen@CasNPs nanoparticles enhanced the permeability across the BBB with NIR light exposure and showed more cytotoxicity toward GBM cells, as well as the GBM stem cell population. Whereas no death was observed in the case of HEK293T cells with and without NIR light exposure (Fig. S5a and b†) indicating the targeting of glioma cells. To confirm the tumor targeting ability of the ICG-Gen@CasNPs, we first tested the selective accumulation of the placebo CasNPs in different cell lines, LN18 and C6. The rhodamine tagged placebo CasNPs showed an enhanced accumulation in the cytoplasm and in the peri-nuclear space in LN18 glioma cells (Fig. 4A) and in C6 cells (Fig. S5c†), but negligible uptake of the nanoparticles was observed in case of the HEK293T cells (Fig. S5d†). The results demonstrated the specificity of the nanoparticles towards the tumor cells compared to normal cells. Our earlier work has shown that nano-curcumin with blue light PDT can effectively inhibit GBM stem cells.^46^ Cellular uptake by monolayer glioma cells may not accurately reflect the delivery efficiency because not only do the nanoparticles have to be taken up by cells but also need to be transported to the deeper tissue regions. In particular for solid tumors, the penetrating ability of nanoparticles is crucial as the interstitial fluid pressures (IFP) are high and there are few vessels in tumor regions. To evaluate the penetration of Cas nanoparticles *in vitro*, multicellular tumor 3D rafts were cultured to mimic the solid tumors.^47,48^ The placebo Cas nanoparticles were incubated with the 3D raft for 4 h. Similar to the observed interactions between the Cas nanoparticles and the cells in the monolayer cultures, the Cas nanoparticles are strongly internalized in the 3D raft (Fig. 4B and C). The 3D raft exhibits a diffused distribution of red color in the peripheral region and the red regions diffuse deeper into the 3D raft. This result suggests that the Cas nanoparticles are able to not only penetrate the cell membrane barriers but also transported in the 3D raft into deeper regions. The penetration of CDDP-loaded Cas nanoparticles into tumor tissue has been demonstrated previously,^49^ verifying that Cas nanoparticles have the ability to diffuse deeper into the 3D raft, this could hold promise for drug delivery *in vivo* in GBM. We concluded that the ICG-Gen@CasNPs acted as an optimized nanosystem to allow transport across the BBB and penetrate into the GBM cells, and that 3D rafts could have applications in the treatment of GBM.

### 3.5 *In vitro* cell cycle and cellular apoptosis analysis

**Fig. 5:**
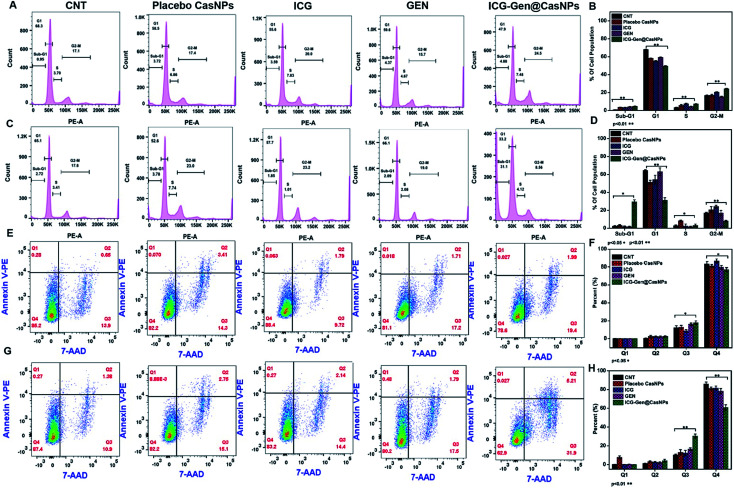
Cell cycle analysis in the LN18 cell line after treatment with Placebo CasNPs, free ICG, GEN, and ICG-Gen@CasNPs with NIR light (A); their quantification (B) and without NIR light (C); and their quantification (D) after 48 h. The cell cycle was analyzed on a minimum of 1 × 10^4^ cells for each experimental condition and data analysis was performed using flow cytometry and Flow Jo analysis software. The quantification into a graph illustrates the percentages of cells in each phase of the cell cycle corresponding to the Sub G1, G1, S and G2M phases of the cell cycle, respectively (A); cell apoptosis determined by Annexin V PE staining and 7AAD. Flow cytometry analysis of the apoptotic and necrotic cells (Q1: dead cells; Q2: early apoptotic; Q3: late apoptotic and necrotic; Q4: live) after 48 h of incubation with the placebo CasNPs, free ICG, GEN, ICG-Gen@CasNPs, without NIR light (E); their quantification (F) and with NIR light treatment (G); and their quantification (H), respectively. The results are expressed as mean ± standard deviation (*n* = 3).

To better characterize the cellular responses, we analyzed the effects of ICG-Gen@CasNPs with NIR light exposure on the cell cycle. The total DNA content-based cell cycle analysis of the ICG-Gen@CasNPs treated glioma cells showed a Sub-G1 phase cell cycle arrest after NIR light exposure compared with the placebo CasNPs, Gen and ICG treated groups. The majority of the cells with ICG-Gen@CasNPs without NIR light treatment (Fig. 5A) are in the G1 phase (47%) and their quantification is shown in Fig. 5B, however with ICG-Gen@CasNPs post-NIR light exposure (Fig. 5C) the accumulation of cells harboring in the Sub-G1 phase (33.6%) can be seen, and their quantification is shown in Fig. 5D showing a marked increase in the apoptotic cells as compared to the control. Sub-G1 phase arrest as an indicator of cell apoptosis has already been investigated.^50^ To analyze the actual mechanism of glioblastoma inhibition we tested whether the ICG-Gen@CasNPs could induce apoptosis in glioblastoma cells. Annexin V-PE/7-AAD based flow cytometry revealed enhanced apoptosis in ICG-Gen@CasNPs (31.9%) (Fig. 5G and H) treated LN18 cells after NIR light exposure which was almost double that found for the ICG-Gen@CasNPs without NIR light treatment (19.6%) (Fig. 5E and F). The overall results compared with ICG, Gen, placebo CasNPs and ICG-Gen@CasNPs with and without NIR light treatment at a predetermined IC_50_ value for 48 h revealed significantly higher apoptosis induction with ICG-Gen@CasNPs after NIR exposure (Fig. 5C). When cells were treated with ICG-Gen@CasNPs and NIR irradiation, the apoptosis level was significantly increased by the combination of the PTT and PDT effect of a previously reported PDT/PTT targeted therapy using gold nanostars in breast cancer.^45^ ICG-Gen@CasNPs with NIR light exposure showed that the majority of the cells did not accumulate in the G2/M phase, but rapidly underwent apoptosis and arrest in the Sub-G1 phase owing to the inability of the cells to cope with the ICG-Gen@CasNPs/NIR induced photodamage. The ICG-Gen@CasNPs combined with NIR light stress increases the photo-damage, which results in prompt activation of the cell death machinery. According to previous reports, the G2/M phase of arrested cells following PDT and photodynamic stress promptly leads to apoptosis. Combinatorial chemo/PDT induces Sub-G1 phase cell cycle arrest *via* multiple controlled pathways.^51^ ICG-Gen@CasNPs act as a smart probe for molecular targeting and therapy. Taking these results together, we conclude that the ICG-Gen@CasNPs multimodal applicator inhibits the proliferation of GBM cells by triggering apoptosis and arresting the cell cycle.

### 3.6 Mitochondrial membrane depolarization and apoptosis analysis

Intracellular ROS generation was recognized as the main mechanism of PDT treatment *via* the apoptosis pathway. Therefore, ROS generation in cells irradiated with free ICG, free GEN, placebo CasNPs, and ICG-Gen@CasNPs, was examined after NIR exposure using a 2′,7′-dichlorofluorescein diacetate (DCFH) ROS detection probe. This is a non-fluorescent compound that can be transformed to be fluorescent (DCFH) *via* ROS-mediated oxidation. Accordingly, the intracellular fluorescence intensity was measured to evaluate ROS generation after a 250 nM (Fig. 7A and B) and a 350 mM dose of ICG-Gen@CasNPs (Fig. 7C and D) with and without NIR light treatment along with the control. It can be seen that there is approximately ten times more ROS generation under NIR light treatment with the 350 nM dose of ICG-Gen@CasNPs compared to the non-radiated dose, signifying the capability of the developed nanoformulation towards cell cytotoxic activity under NIR exposure, which is supported by previous reports^52^ confirming ROS generation using PDT therapy.

**Fig. 6:**
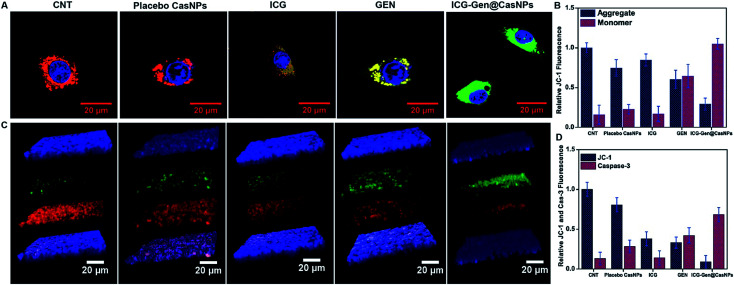
Confocal light scanning microscopy imaging showing the JC 1 expression in LN18 glioblastoma cells after treatment with free placebo CasNPs, ICG, GEN, and ICG-Gen@CasNPs with NIR light treatment (A); their relative fluorescence quantification is shown in (B). Z-stack images of JC 1 and caspase 3 in LN18 in 3D rafts (C); their relative florescence is shown in (D). ICG-Gen@CasNPs shows apoptosis effects increase in caspase 3 as compared to others, showing the increased expression of caspase 3 and decreased expression of JC 1 in the rafts.

**Fig. 7:**
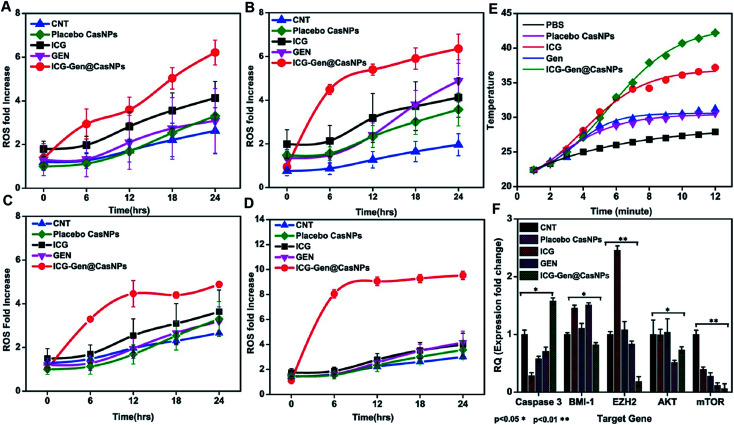
ROS assays with different doses of GEN, ICG-Gen@CasNPs, ICG, placebo CasNPs, with a 250 nM dose without NIR light (A),with NIR light exposure (B), and a 350 nM dose of GEN, ICG-Gen@CasNPs, ICG, placebo CasNPs without NIR light (C), with NIR light (D); temperature changes in response to 808 nm laser irradiation with a power density of 0.6 W cm^−2^ in PBS, placebo CasNPs, ICG,GEN, ICG-Gen@CasNPs (E); QPCR analysis for caspase 3, BMI-1, EZH2, AKT, mTOR with the control, ICG, GEN, placebo CasNPs, ICG-Gen@CasNPs with NIR light exposure (F).

The previous reports confirmed the involvement of ROS in the induction of apoptosis and demonstrated the importance of ROS in the mitochondria directed by the apoptosis process.^53^ We evaluated the mitochondrial dysfunction by using a mitochondrial membrane potential sensitive dye, JC-1. The results further proved that after NIR light exposure ICG-Gen@CasNPs induce the depolarization of the mitochondrial membrane potential and induce a higher mitochondrial potential loss, as confirmed using a confocal imaging. The untreated controlled cells produce a red fluorescence as the JC-1 aggregates are converted into a monomer and exhibit a green fluorescence upon depolarization in the LN18 cells after NIR light exposure with ICG-Gen@CasNPs (Fig. 6A and B) as compared to others groups showing the high potency of the ICG-Gen@CasNPs, which can efficiently increase the level of intracellular ROS in the mitochondria and further apoptosis occurs. Furthermore, the mitochondrial function was also analyzed in LN18 three-dimensional raft culture models after ICG-Gen@CasNPs treatment after NIR light exposure. We found a significant increase in the caspase-3 expression level green fluorescence by ICG-Gen@CasNPs as compared to other groups, indicating that the enhanced caspase-3 dependent apoptosis could be correlated with the increased ROS generation and apoptosis in the LN18 three dimensional rafts (Fig. 6C and D). The result suggested that ICG-Gen@CasNPs with NIR exposure causes depolarization of the mitochondrial membrane which is clearly linked with the induction of apoptosis, the increased expression of caspase-3 and increased ROS generation in the glioma. The results revealed that the preliminary mechanism of tumor cell death was induced by the high ROS generation, severe damage to the mitochondrial membrane potential, and that these finally lead to apoptotic cell death. Some literature has also reported ROS generation induced using 808 nm NIR light. Mitochondria regulate energy metabolism and are intimately affected by excessive generation of ROS, which results in a change in the mitochondrial membrane potential.^54^ In the case of silica-Ce6-FA/PDT treated MDA-MB-231 cells, the loss of the mitochondria membrane potential with mitochondrial damage has been reported.^55^ In our results, the ICG-Gen@CasNPs have shown great potential for use as a nano-photosensitizer targeted towards the mitochondria and can induce a mitochondrial ROS burst in GBM cells. The excessive formation of ROS under PDT therapy leads to mitochondrial dysfunction that could cause ATP depletion and further induces cell death through apoptosis.

We also evaluated the PTT properties of ICG-Gen@CasNPs by measuring the temperature changes of ICG-Gen@CasNPs at different concentrations under the irradiation of an 808 nm laser (0.6 W cm^−2^) (Fig. 7E). PBS was used as a control. The temperature of the ICG-Gen@CasNPs with NIR light exposure in solutions increased rapidly within 15 min and gradually reached 42 °C, indicating that the ICG-Gen@CasNPs can quickly convert laser energy into environmental heat which can sufficiently kill the tumor tissue and prevent GBM reoccurrence. Previously published studies report the killing of tumor cells at temperatures of 42–43 °C.^56^ In contrast, PBS showed negligible temperature changes under the same NIR exposure conditions, indicating that these NIR exposure conditions cause a minimal risk of adversely affecting cells or tissues. Notably, the PDT/PTT effect of ICG-Gen@CasNPs elicits a chemotherapeutic effect synergizing multiple pathways.

Overall, the combined therapy of photodynamic and photothermal ablation and chemotherapy can synergistically improve the cancer therapeutic efficiency of the ICG-Gen@CasNPs, providing a novel drug delivery system for GBM therapy.

### 3.7 Gene expression profiling and western blot expression analysis

Upon analyzing the fold change in gene expression using real-time PCR, an approximately 1.5 fold increase of the caspase 3 mRNA level was observed after a predetermined IC_50_ dose of ICG-Gen@CasNPs for treatment up to 48 h under light exposure (*n* = 3). The downregulation of the Akt (∼0.5 fold) and mTOR (∼0.1 fold) expression (Fig. 7F) was consistent with the earlier reported partially known molecular mechanism of Gen action in breast cancer^57^ and indicated the enhanced bioavailability of Gen and that the mechanism of the chemotherapeutic effect and the decreased proliferative potential of the glioma leads to retarded cancer progression.

**Fig. 8:**
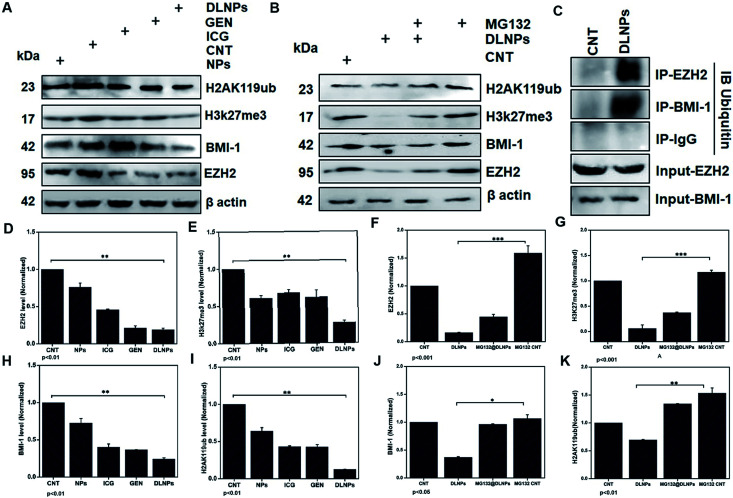
Western blot analysis after treatment with NPs (placebo CasNPs), ICG, Gen, DLNPs (ICG-Gen@CasNPs) with NIR light exposure for beta actin, EZH2, BMI-1, H3K27me3, H2AK119ub (A); and their quantification for EZH2 (D), H3K27me3 (E), BMI-1 (H), H2AK119ub (I); western blot analysis co-treatment of MG132 and ICG-Gen@CasNPs treatment (B); and their quantification for EZH2 (F), H3K27me3 (G), BMI-1 (J), H2AK119ub (K). The beta-actin level was measured to normalize loading. GBM cells were treated as above, and the total extracts were immunoprecipitated with anti IgG, anti-EZH2, and anti-BMI1. The precipitates were then electrophoresed and immunoblotted to detect ubiquitin and the western blot was analyzed using ubiquitin (C).

EZH2 has been implicated in stem cell maintenance and is overexpressed in different cancers including malignant gliomas, and the silencing of EZH2 reduced glioma cell proliferation and invasiveness.^58^ Epigenetic regulators including BMI1 and EZH2 have already been investigated in gliomas and new strategies are being tested, such as the inhibition of EZH2, a histone methyltransferase which is overexpressed in glioma cells, leading to angiogenesis and metastasis.^59^ We analyzed the polycomb expression in the LN18 GBM cell line after treatment with ICG-Gen@CasNPs with NIR light exposure. Our results showed a decreased level of BMI-1 (∼0.8) and EZH2 (∼0.1) compared to the untreated control in real time PCR, indicating the NIR light induced regulation of polycomb gene expression. Furthermore, downregulation of PRC2 (EZH2) (Fig. 8A–D) and the PRC1 (BMI-1) (Fig. 8A–H) protein level after treatment of ICG-Gen@CasNPs with NIR light exposure in western blot confirmed the role of PcG in the regulation of glioma survival. The decreased protein level of H3K27me3 (Fig. 8A–E) and H2AK119ub (Fig. 8A–I) in the western blot analysis shows the mutual role of the PRC1 and PRC2 complex in glioblastoma progression. EZH2 trimethylates histone H3 at lysine 27 and recruits members of BMI-1 having E3 ligase activity for the monoubiquitination of histone H2A at lysine 119, which results in transcriptional repression of the tumor suppressor genes.^60^ A functionalized upconversion nanoparticle (UCNP)-based drug delivery system which can target brain tumors and convert deep tissue-penetrating NIR light into visible light for phototherapy has already been reported^61^ in a previous study, but until now no reports have been published regarding the PDT/PTT therapy of nanoparticle targeting polycomb protein expression in GBM. Thus, in our studies we confirm the role of the polycomb group of the protein mediating the anti-glioblastoma effect of the ICG-Gen@CasNPs in response to the use of chemo/PDT/PTT therapy. For the first time, our nanoformulation ICG-Gen@CasNPs demonstrate the role of combinational chemo/PDT/PTT therapy in correlation with the polycomb group of proteins and also significantly reduced polycomb expression, demonstrating a new strategy for the treatment of glioma. Previous studies have reported that the dual targeting strategy of EZH2 and BMI-1 may be useful in combination against glioma stem cells, suggesting that simultaneously they can target multiple epigenetic regulators within glioblastomas.^59^

Furthermore, to determine the mechanism of downregulation of the polycomb group of proteins we performed the immunoprecipitation (IP) of EZH2 and BMI-1 and western blot with anti-ubiquitin (Ub) after treatment with ICG-Gen@CasNPs and after NIR light irradiation indicated the associated polyubiquitination of cellular EZH2 and BMI-1 (Fig. 8C). The continuous trailing in ICG-Gen@CasNPs after NIR light exposure represents the degree of ubiquitination and temporal degradation. The analysis of results showed enhanced polyubiquitination of EZH2 and BMI-1 in ICG-Gen@CasNPs with exposure to NIR light in comparison to the untreated control. Also, IP with anti-IgG showed no association of ubiquitin in western blot analysis suggesting assay specificity. The ubiquitination directs the protein toward proteasomal degradation and maintains the functional homeostasis of the cells. The intracellular mechanism of the PcG protein suppression by ICG-Gen@CasNPs is not well understood. The major finding is the downregulation of Ezh2 expression and a key question is the mechanism of EZH2 downregulation. Furthermore, to confirm the mechanism of inhibition of proteasome function, the proteasomal pathway inhibitor (MG132) was used which effectively blocks the proteolytic activity of the proteasomal complex. In our results, the inhibitory effect of ICG-Gen@CasNPs on EZH2 (Fig. 8B–F), BMI-1 (Fig. 8B–J), H3K27me3 (Fig. 8B–G), and H2AK119ub (Fig. 8B–K) was lost after co-treatment with a 2.5 μM dose of MG132 with NIR light indicating the crucial role of polyubiquitination and proteasome-mediated degradation. Our results are similar to another report showing that DZNep promotes proteasome-dependent EZH2 degradation and that the DZNep-dependent reduction in EZH2 level is associated with reduced H3K27me3 formation, which is an EZH2-specific histone modification.^62^ In our results the increased polyubiquitination of EZH2 and BMI-1 after ICG-Gen@CasNPs with NIR light exposure indicates the superior targeting of the PcG complexes by eliciting proteasome degradation. The present study demonstrated that the novel multimodal ICG-Gen@CasNPs system inhibits the expression of EZH2 in LN18 human glioma cells by chemo/PDT/PTT therapy. These findings suggested that ICG-Gen@CasNPs could be a potentially promising therapeutic target for polycomb expression in GBM treatment.

### 3.8 Epigenetic regulation of oxidative stress as a critical mediator of the anti-GBM effect

**Fig. 9:**
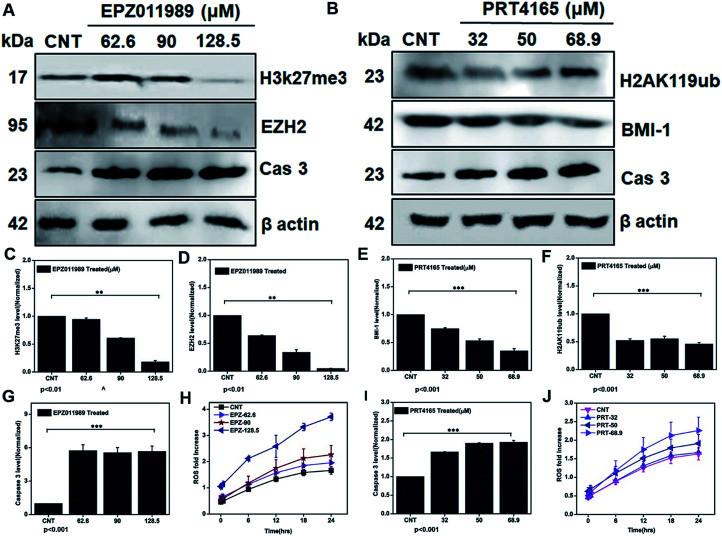
Western blot analysis after treatment with EPZ011989 (EZH2 inhibitor) for beta actin, Cas 3, EZH2, H3K27me3 (A) and their quantification for H3K27me3 (C), EZH2 (D), Cas 3 (G), and ROS analysis after treatment with different EPZ011989 doses of EPZ (H). Western blot analysis after treatment with PRT4165 (BMI-1 inhibitor) for beta actin, Cas 3, BMI-1, H2AK119ub (B) and their quantification for BMI-1 (E), H2AK119ub (F), and caspase 3 (I). ROS estimation after treatment with a dose of PRT4165 (J).

To further demonstrate that depleting the polycomb group of proteins in the subunits EZH2 and BMI-1 leads to ROS generation and mediating apoptosis we treated the glioblastoma cells with IC_25_, IC_50_, and IC_75_ doses of the EZH2 (EPZ011989) and BMI-1 inhibitor (PRT4165). Downregulation of the EZH2 (Fig. 9A–D), H3K27me3 (Fig. 9A–C), BMI-1 (Fig. 9B–E), and H2AK119ub (Fig. 9B–F) protein level showed that the inhibitory effect of the drugs correlated with the increased ROS (Fig. 9H), and that caspase 3 (Fig. 9A–G) with the EZH2 inhibitor and the BMI-1 inhibitor increased the ROS (Fig. 9J) and caspase 3 (Fig. 9B–I) cellular expression. Thus, our investigation reveals that targeting polycomb repressor proteins could significantly elicit ROS cellular stress and apoptosis-related proteins in GBM. Moreover, the increased ROS stress in the context of the DZNep-mediated depletion of EZH2 and H3K27me3 in acute myeloid leukemia has already been reported.^63,64^ BMI-1 is functionally linked to the oxidative metabolism of cancer cells and confers resistance to oxidative stress and downregulation promotes a damaging effect. Taken together these results indicate the role of the PRC subunits in the regulation of the cellular stress mechanism. Several studies demonstrated that dysregulation of intracellular ROS damages self-renewal and leads to stem cell failure.^65^ This inhibition of the expression of EZH2 and BMI-1, in turn, led to apoptosis *via* the generation of ROS in GBM. The multimodal targeting of PcG with ICG-Gen@CasNPs could lead to accumulation of cellular ROS, activation of programmed cell death and therefore counteracts GBM growth. Previously reported studies have shown the link between epigenetic regulation and oxidative stress, but for the first time, we have shown the role of BMI-1 and EZH2 mediated ROS generation and their link with our nanoformulation ICG-Gen@CasNPs chemo/PDT/PTT therapy. Unlike chemotherapy, PDT offers better post-therapeutic quality of life and has no known side effects. The use of the PDT effect in breast and cervical cancer stem cells using low-intensity laser irradiation for effective treatment could be a potential therapeutic tool, given its ability to trigger apoptotic cell death.^66^ Taken together, our work uncovers the novel mechanism of ICG-Gen@CasNPs/NIR induced anti-GBM efficacy by targeting PcG and provides a strong rationale to further develop strategies to target PcG in GBM. These novel findings provide further insights into the epigenetic links between oxidative stress and highlights a crucial target for anti-glioblastoma therapy.

### 3.9 Trans-well assay and permeability assessment

A primary endothelial cell monolayer cultured in coated Transwell inserts mimicking the real BBB architecture enabled us to investigate the ability of CasNPs to cross this barrier. In addition, the mechanism of the junction permeability after CasNPs interactions and their potential to pass through are the basis of the permeability.^67^ The tight junction integrity defines the regulation of the monolayer permeability in the BBB. The permeability across the endothelial cell monolayer was measured using the fluorescence of CasNPs in the lower chamber (brain side) as a function of time. The endothelial cell permeability is higher in the initial hours and at a maximum up to 12 h (36%), after which it starts to steadily decrease up to 48 h (Fig. S6†). Moreover, there was no constraints with the insert as it did not contain a cell monolayer as a control. The mechanism reported in the literature described changes in the F-actin and vascular endothelial cadherin which triggered the rearrangement of the tight junction and the adherence junction allowing passage through the membrane.^68^ Thus, the ability of the CasNPs to cross the *in vitro* endothelial cells demonstrated the permeabilization potential of the BBB.

### 3.10 *In vivo* and *ex vivo* bio-distribution of nanoparticles and therapeutic assessment

**Fig. 10:**
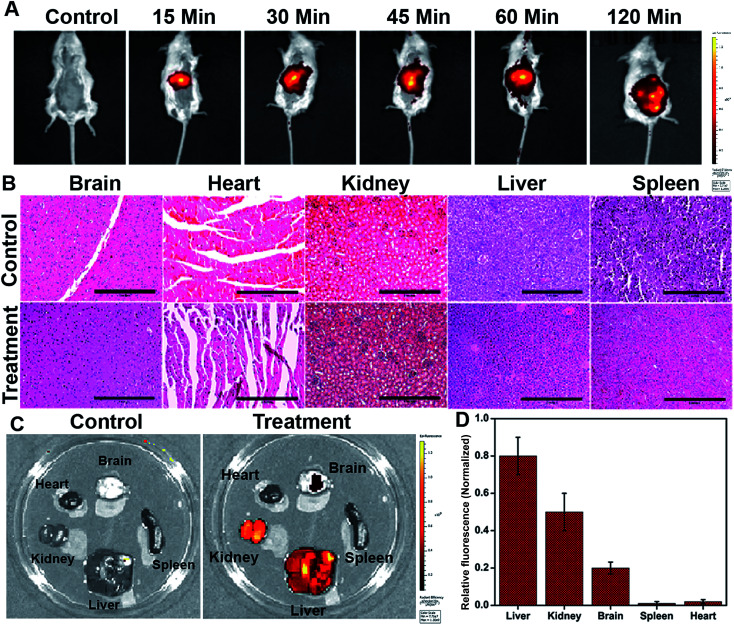
Biodistribution of ICG-Gen@CasNPs nanoparticles tagged with ICG used for whole animal imaging 15, 30, 45, 60, and 120 min post intravenous injection (A); *in vivo* toxicology evaluated *via* histological staining of heart, kidney, liver, spleen and brain sections *via* hematoxylin and eosin staining (scale bar: 1 inch) (B); *ex vivo* imaging of organs harvested after injection (C); the organs (from top left to bottom right) are brains, heart, liver, kidney, and spleen; the quantification of relative florescence (D).

**Fig. 11:**
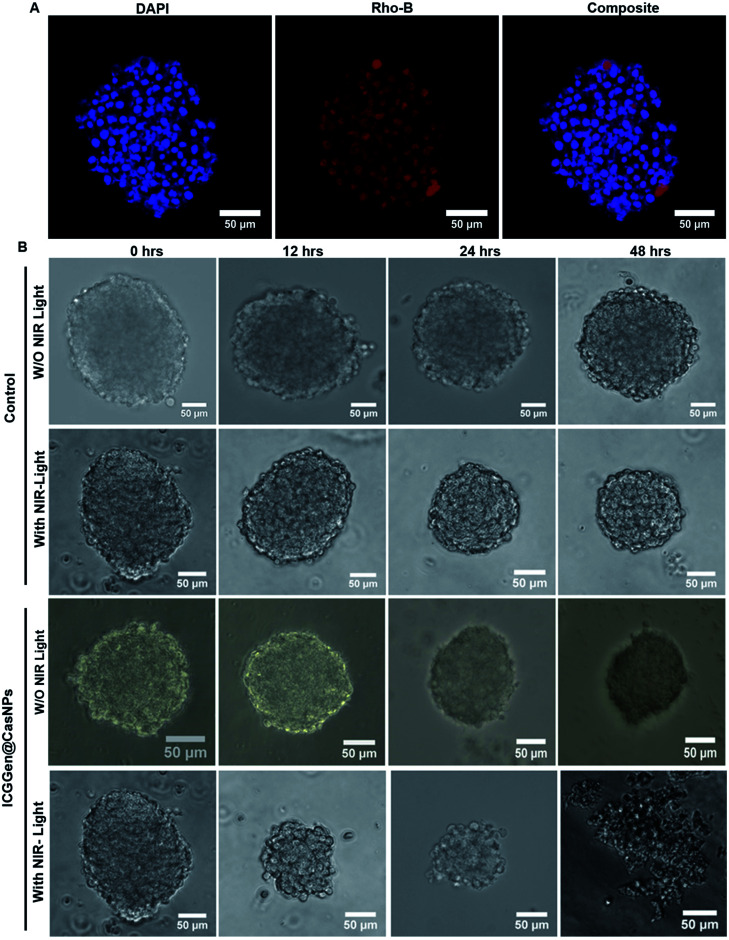
Confocal microscopy images showing the uptake in LN18 spheroids treated with rhodamine B tagged ICG-Gen@CasNPs (A); *in vitro* therapeutic assay in LN18 spheroids with ICG-Gen@CasNPs with NIR light and without NIR light exposure (B). All scale bars are 50 μm.

The *in vivo* whole-body distribution of the fluorescently (ICG) tagged ICG-Gen@CasNPs was assessed by injection into BALB/c mice *via* the tail vein, and observation of the photoluminescence (IVIS spectrum) of the ICG using NIR fluorescence imaging was observed for 4 h at time intervals of 30 min (Fig. 10A). ICG tagged ICG-Gen@CasNPs showed a wide distribution pattern with limited accumulation in a period of 4 h with an initial gathering in the liver and kidney after this time. The *ex vivo* distribution was observed *via* photoluminescence in the kidney, spleen, liver, brain, and heart (Fig. 10C). The *ex vivo* bioimaging, in which the liver and kidney show major accumulation of the ICG-Gen@CasNPs after the observation time reconfirms the systemic and visceral clearance of the nanoformulation and accumulation of ICG-Gen@CasNPs in brain confirm that it crosses the BBB and overcomes the main obstacle for the treatment of glioma by sufficient exposure of the drugs at the site of action within the brain. The BBB causes major problems in the therapy of brain tumors because it prevents the delivery of most drugs and targeted agents to the tumor location and different strategies have been investigated to facilitate the BBB crossing of therapeutics,^69^ such as the use of conjugated or surface functionalized nanocarriers which can cross the BBB. In our study, a single multimodal application of ICG-Gen@CasNPs demonstrates the targeted delivery of Gen by crossing the BBB, giving hope for the treatment of GBM. *In vivo* toxicology evaluated *via* the histological staining of the heart, kidney, liver, spleen and brain sections *via* hematoxylin and eosin staining (Fig. 10B), shows negligible toxicity in the heart, kidney, liver, spleen and brain tissues in comparison to the control group implying the low toxicity and good biocompatibility of ICG-Gen@CasNPs *in vivo*. The fluorescence of ICG in the ICG-Gen@CasNPs was quantitated by observing fluorescence in the thin section of organs. The maximum accumulation in the liver (∼80%), Kidney (56%) and brain (19%) observed was compared to the untreated control sections (Fig. 10D). The spleen and heart show negligible accumulation of particles. Thus, the results confirm the accumulation of the ICG-Gen@CasNPs in the brain and indicating the BBB crossing ability. IV administration is favored for systemic drug delivery as it bypasses many of the absorption barriers, efflux pumps, and metabolic mechanisms. Direct administration of a drug into the vascular space enables maximum bioavailability with an effective and constant level of the drug in the bloodstream. In particular, the advantages of CasNPs IV administration were evident in the rat model. The drug-loaded Cas nanoparticles exhibited a prolong circulation time with markedly less systemic clearance. The pharmacokinetic studies revealed that CasNPs as a drug carrier increases the half-life of the drug from 0.88 to 14.65 h and it is 12.6-fold less susceptible to the reticuloendothelial system (RES). In addition, the systemic delivery of a particle diameter of less than 200 nm displayed a prolonged blood resident time probably owing to reduced susceptibility to the opsonization process and reduced uptake by the RES system minimizing the non-specific toxicity associated with the hepatic system. The presence of the hydrophobic domain in the proteins plays a major functional role in retaining the drug and also controlling the release rate. These characteristics realize the use of the protein-based carrier for a drug delivery vehicle for precise delivery at the disease site. The ICG-Gen@CasNPs nanoformulation for targeting the brain and NIR imaging applications can be further used in image-guided therapy in GBM. The therapeutic assuagement on 3D spheroids with or without ICG-Gen@CasNPs following NIR was evaluated (Fig. 11). Spheroids were formed containing LN18 tumor cells as *in vitro* model mimicking real human tumor architectures. Primarily, the ability of rhodamine tagged ICG-Gen@CasNPs for penetrating solid spheroids was assessed by performing spheroid uptake experiments under CLSM. The particles showed a good penetration ability in 3D spheroids and this was confirmed in the 3D raft uptake model (Fig. 11A). After formation spheroids were washed and transferred to new wells, exposure to ICG-Gen@CasNPs completely inhibited their growth following NIR over 48 h (Fig. 11B) compared to the non-treated controls or NIR treated controls. The temporal effect of ICG-Gen@CasNPs with NIR, along with the control, was quantitatively drawn as a function of the spheroid diameter and time of treatment, representing complete disintegration of a solid tumor at a time point of 48 h (Fig. S7†). Whereas a steady decrease in the spheroid diameter was observed upon ICG-Gen@CasNPs treatment. Thus, this simple *in vitro* spheroid model eliminates many of the problems encountered with the monolayer or animal studies and mimics micro-tumors structures before vascularization. Advantageously, tumor cells within spheroids represent a higher degree of morphological and functional differentiation than the same cells grown in monolayer cultures. The complex response of growth kinetics, metabolic rates, resistance to radiotherapy and photodynamic and chemotherapy are similar to tumor cells *in vivo*.^70^

The combination of chemo/PDT is regulating multiple mechanisms for chemoprevention, including signal transduction mediated by generated ROS, enhanced apoptosis, overexpression of caspase-3, Sub-G1 cell cycle arrest for abrogating glioblastoma proliferation. The present therapy can provide extended protection against tumor recurrence by killing tumor stem cell populations. Furthermore, gene expression analysis revealed that PDT compliments the chemotherapeutic effect of ICG-Gen@CasNPs in a controlled manner. The significant downregulation of the polycomb group of genes (EZH2, BMI-1) indicates that reduced global histone methylation could be reverting tumor suppressor gene silencing by reducing promoter methylation and histone modifications. The nanoformulation triggered ubiquitin-mediated proteasomal degradation of the polycomb protein produces greater ROS generation augmenting GBM cell death. Further studies could elaborate on the functional relevance of combination chemo/PDT induced epigenetic regulation of chemo-preventive efficacy in GBM.

## 4. Conclusions

In this study, ICG-Gen@CasNPs combining a photo/chemo dual therapeutic efficacy were established as a potential inhibitor of PcG in GBM. The fabricated nanosized ICG-Gen@CasNPs exhibit eminent monodispersity, stability, and accumulation in the GBM monolayer and 3D cultures. The NIR responsive ROS generation, apoptotic pathway activation, cell cycle arrest, and mitochondrial membrane depolarization were linked to the efficacy of ICG-Gen@CasNPs. ICG-Gen@CasNPs/PDT triggered suppression of PcG by directing the ubiquitin-mediated degradation of the major PcG proteins. The role and precise mechanism of the polycomb group of proteins upon ICG-Gen@CasNPs/PDT were linked for the first time with ROS generation and apoptotic pathways in the glioblastoma cells. Thus, the proteasomal degradation of the polycomb group of proteins in response to chemo/photodynamic therapy revealed that the PcG mediated regulation of oxidative stress can be used as a novel target in GBM therapy.

## Conflicts of interest

There are no conflicts to declare.

## Supplementary Material

NA-001-C9NA00212J-s001
