# Supplementary material for: A NIR-responsive indocyanine green-genistein nanoformulation to control the polycomb epigenetic machinery for the efficient combinatorial photo/chemotherapy of glioblastoma
Source: Nanoscale Adv. 2019 Apr 16;1(6):2188–207. doi: 10.1039/c9na00212j (PMC9419092; doi:10.1039/c9na00212j)
Supplement: NA-001-C9NA00212J-s001 [file NA-001-C9NA00212J-s001.pdf]

Electronic Supplementary Material (ESI) for Nanoscale Advances.
This journal is © The Royal Society of Chemistry 2019

Supporting information

NIR-responsive Indocyanine Green-Genistein Nanoformulation Controlling Polycomb Epigenetic Machinery for Efficient Photo-Chemo-Combotherapy of Glioblastoma

Babita Kaundal,[a] Anup K. Srivastava,[a] Mohammed Nadim Sardoiwala,[a] Surajit Karmakar[a] and Subhasree Roy Choudhury,*[a]

Run	Protein conc. (%)	Acetone conc. (%)	Stirring rate (rpm)	Particle size (nm)	PDI
**1**	1.00	90.00	1200.00	206.5	0.43
**2**	1.00	80.00	1800.00	192.74	0.35
**3**	1.50	90.00	1800.00	156.86	0.27
**4**	0.50	80.00	1200.00	236.1	0.51
**5**	1.00	80.00	600.00	185.76	0.35
**6**	1.00	100.00	1800.00	127.1	0.35
**7**	1.00	90.00	1200.00	211.86	0.37
**8**	1.00	100.00	600.00	173.66	0.34
**9**	1.00	90.00	1200.00	211.4	0.38
**10**	1.50	90.00	600.00	232	0.32
**11**	1.50	100.00	1200.00	197.08	0.36
**12**	0.50	100.00	1200.00	143.16	0.43
**13**	1.00	90.00	1200.00	211.4	0.38
**14**	0.50	90.00	1800.00	198.06	0.43
**15**	0.50	90.00	600.00	160.46	0.41
**16**	1.50	80.00	1200.00	259.06	0.39
**17**	1.00	90.00	1200.00	226.03	0.42

Table S1: Reaction trials with the variables

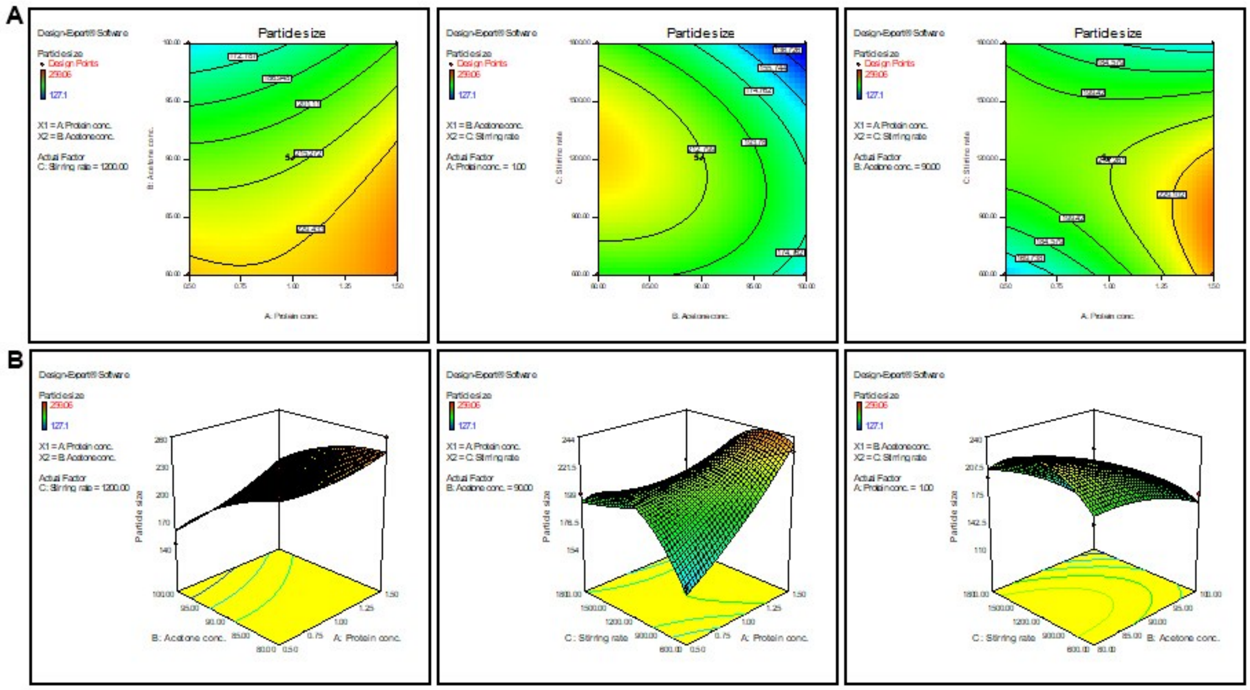

Figure. S1 2D Contour plots showing pattern among the variables(A); 3D response plots showing interaction among the variables(B).

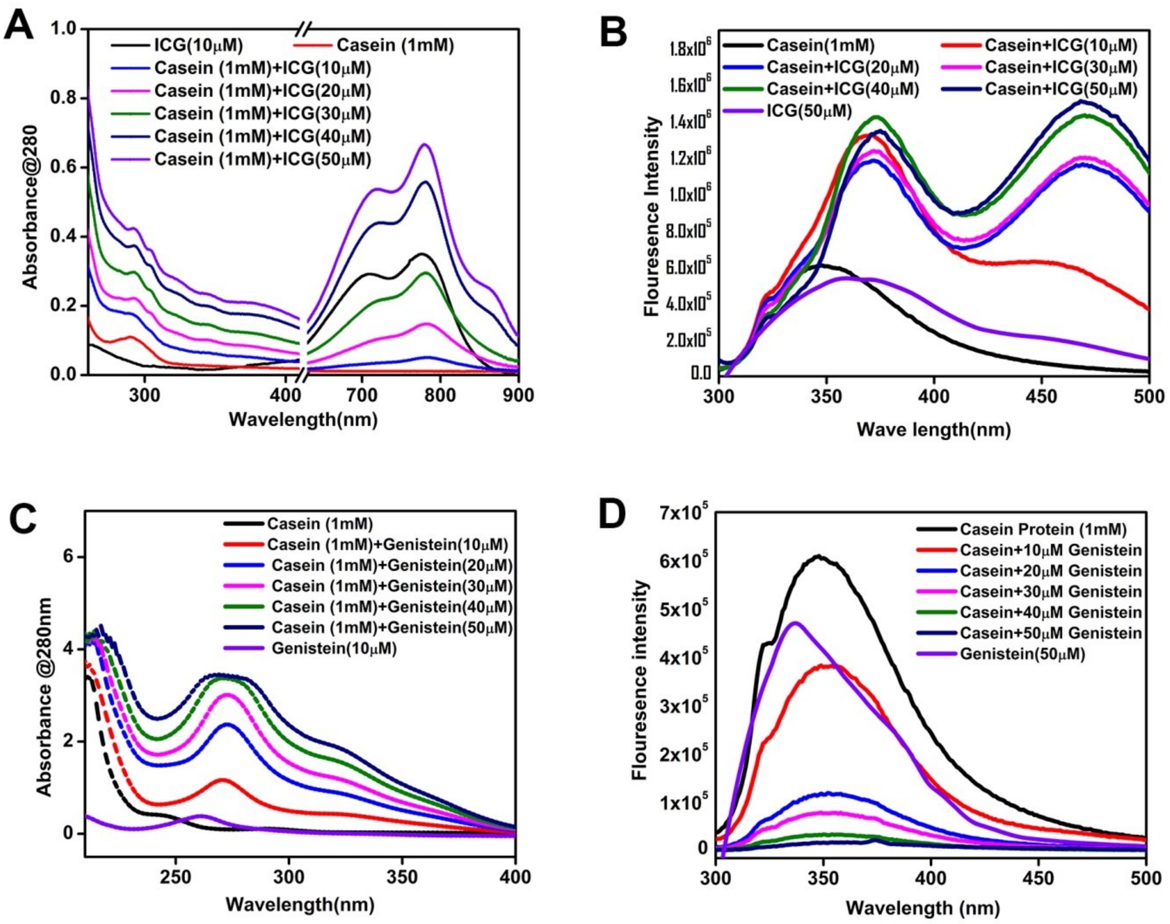

Figure S2. UV Visible Spectroscopic data representing the physical interactions of casein and ICG , casein and genistein enhancing the native absorbance of protein at λ_{max}=280 nm (Shown via black arrow) (A and C); fluorescence spectroscopy data representing the physical interactions of casein and ICG, Casein and genistein quenching the native Florescence of protein at λ_{max}=280 nm (B and D).

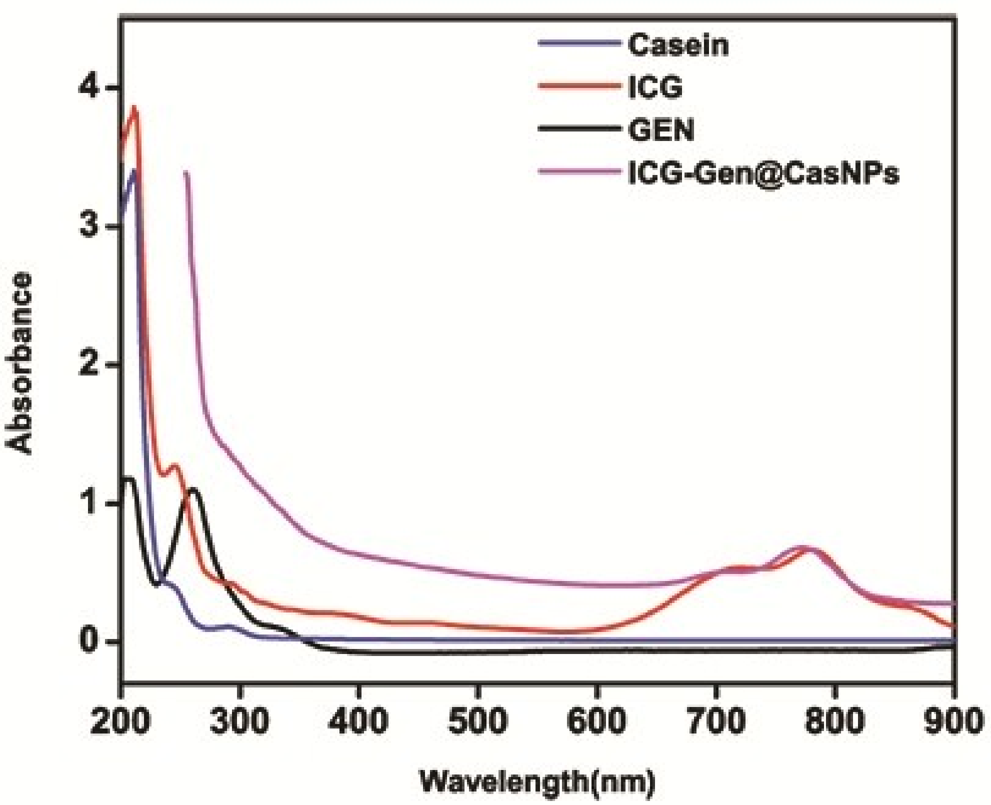

Figure S3. UV-Vis spectra of Casein, ICG, GEN and ICG-Gen@CasNPs**.**

.

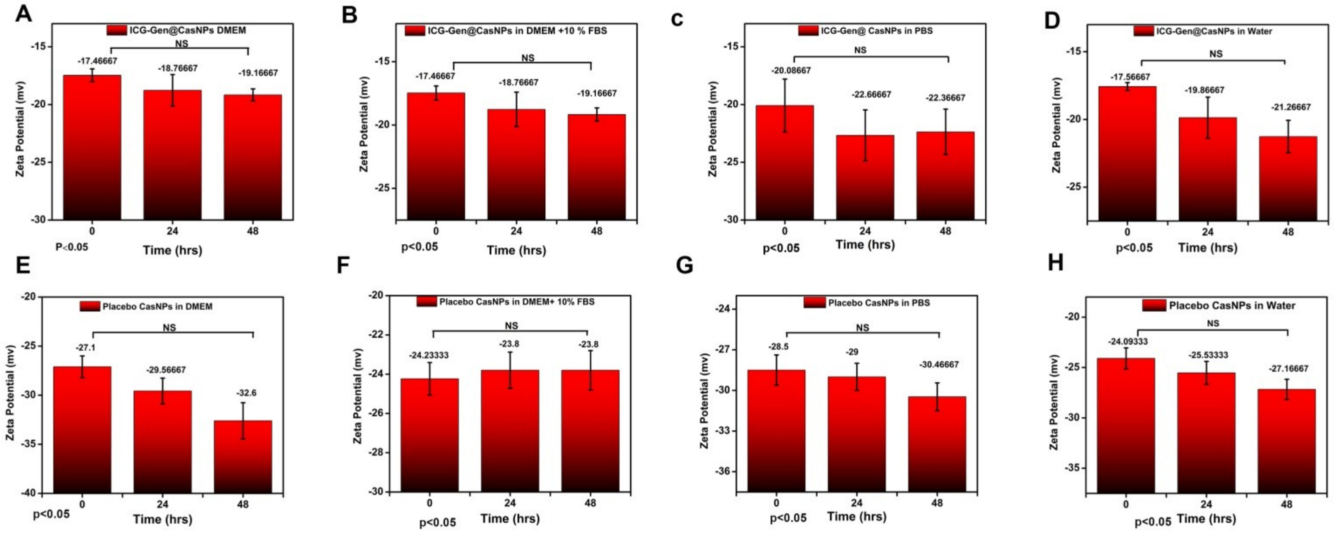

Figure S4 The histogram showing the Zeta potential value of ICG-Gen@CasNPs (A,B,C,D) and Placebo CasNPs (E,F,G,H) in DMEM and DMEM +10% FBS , PBS, Water respectively. The zeta potential statistically same till 48 hrs observed via one way ANOVA with Tukey test at significant level of P≤0.05 (n=3).

.

.

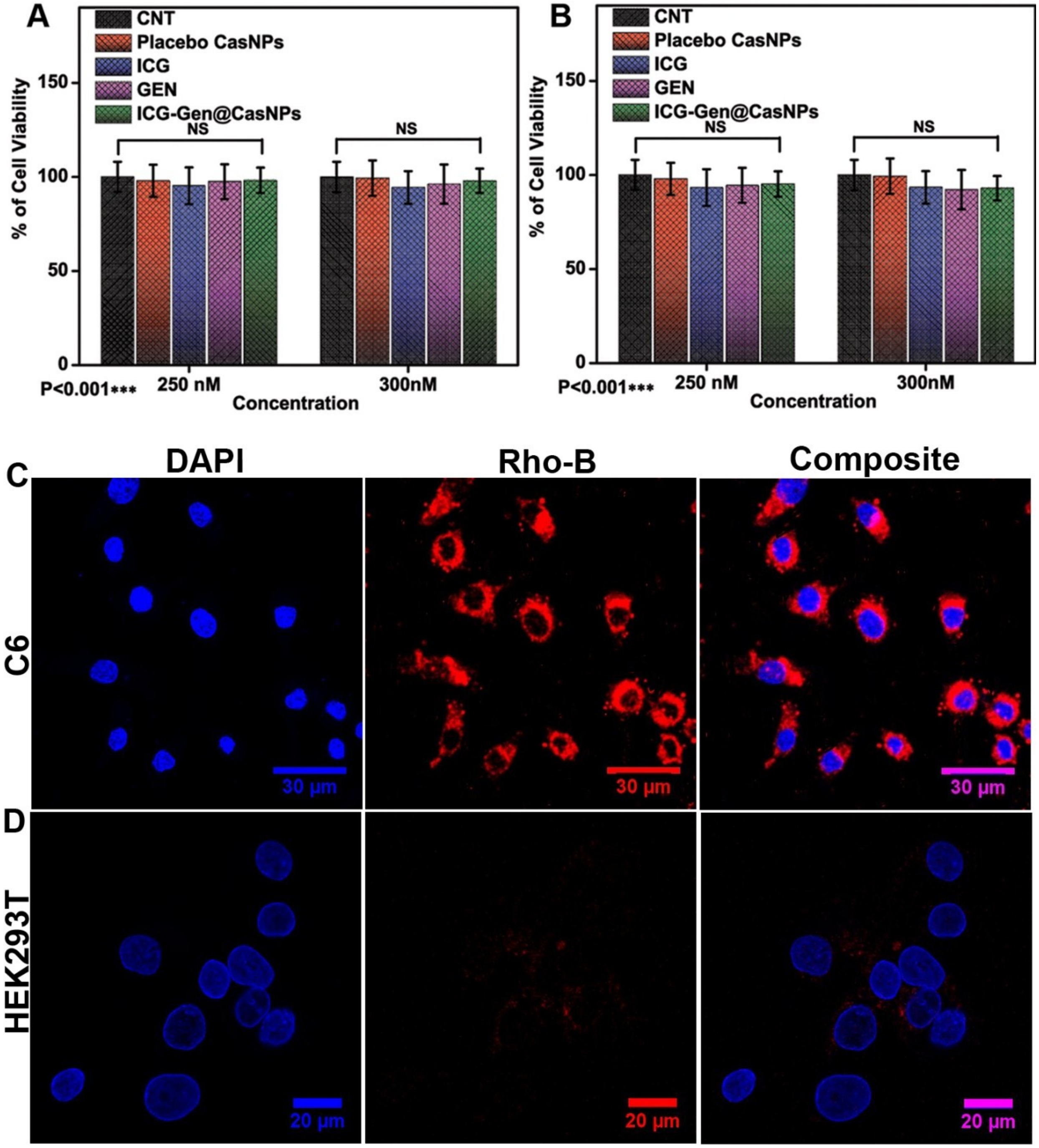

Figure S5. Cellular viability assay showing in HEK293T cells with different dose control, placebo CasNPs, Genistein, ICG, ICG-Gen@CasNPs without NIR light (A) and with NIR light

(B); Cellular uptake of Rhodamine B tagged Nanoparticle showing in C6 cell line (C); and in HEK293T(D).

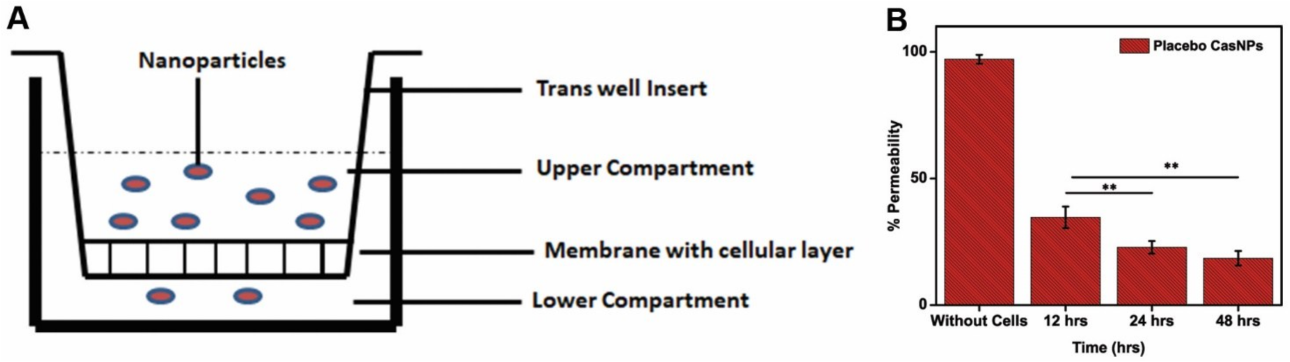

Figure S6. The *in-vitro* trans well model mimicking BBB and pemeability assay of ICG-Gen@CasNPs. (A) representation of basic procedure of trans well seperating luminal side (luminal side) and brain side (lower chamber) seperated by endothelial cell monolayer. (B) the normalized fluorescence intesity of the particle present in the lower chamber at 12 hr, 24hr and 48 hr relative to particles passed through insert cotain no cells.

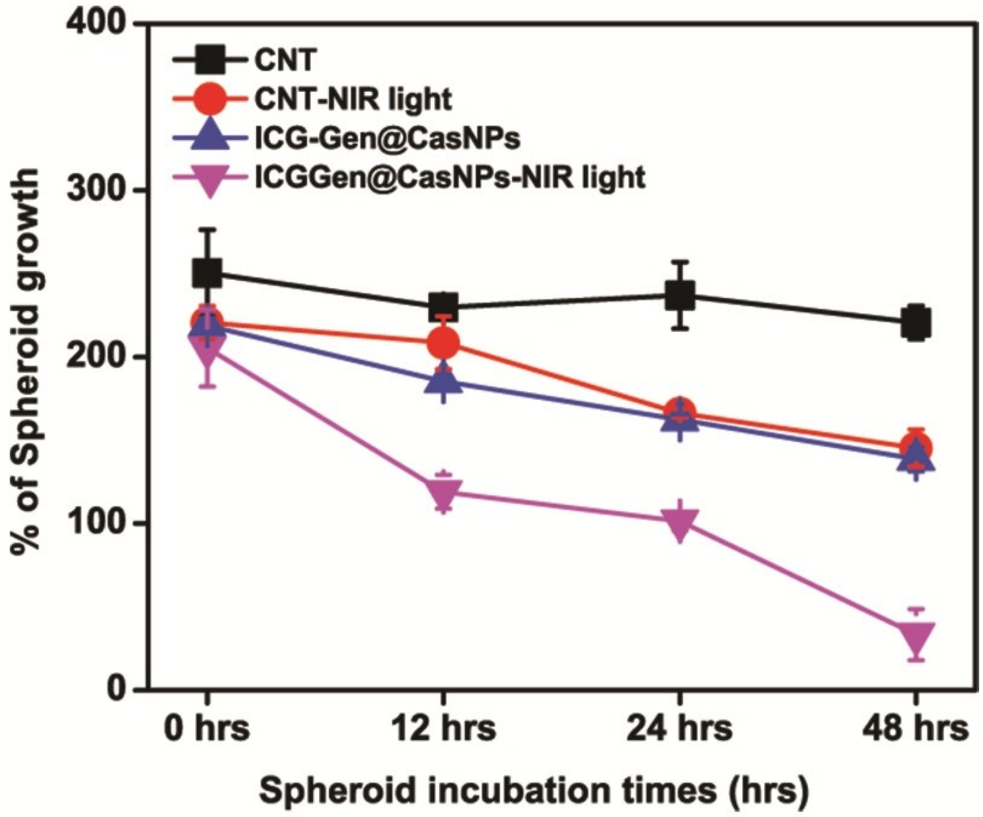

Figure S7. The *in-vitro* 3D glioma spheroids treated with ICG-Gen@CasNPs and ICG-Gen@CasNPs+NIR idnicating excellant disruption in 48 hrs.
